# Modeling and Predicting Outcomes of eHealth Usage by European Physicians: Multidimensional Approach from a Survey of 9196 General Practitioners

**DOI:** 10.2196/jmir.9253

**Published:** 2018-10-22

**Authors:** Joan Torrent-Sellens, Ángel Díaz-Chao, Ivan Soler-Ramos, Francesc Saigí-Rubió

**Affiliations:** 1 Faculty of Economics and Business Universitat Oberta de Catalunya Barcelona Spain; 2 Applied Economics Department King Juan Carlos University Madrid Spain; 3 Faculty of Health Sciences Universitat Oberta de Catalunya Barcelona Spain

**Keywords:** internet, eHealth, health care, health drivers, health barriers, health attitude, health information, health empowerment, information and communication technologies, structural equation modeling, Europe

## Abstract

**Background:**

The literature has noted the need to use more advanced methods and models to evaluate physicians’ outcomes in the shared health care model that electronic health (eHealth) proposes.

**Objective:**

The goal of our study was to design and evaluate a predictive multidimensional model of the outcomes of eHealth usage by European physicians.

**Methods:**

We used 2012-2013 survey data from a sample of 9196 European physicians (general practitioners). We proposed and tested two composite indicators of eHealth usage outcomes (internal practices and practices with patients) through 2-stage structural equation modeling. Logistic regression (odds ratios, ORs) to model the predictors of eHealth usage outcomes indicators were also calculated.

**Results:**

European general practitioners who were female (internal practices OR 1.15, 95% CI 1.10-1.20; practices with patients OR 1.19, 95% CI 1.14-1.24) and younger—aged <35 years (internal practices OR 1.14, 95% CI 1.02-1.26; practices with patients OR 1.32, 95% CI 1.13-1.54) and aged 36-45 years (internal practices OR 1.16, 95% CI 1.06-1.28; practices with patients OR 1.21, 95% CI 1.10-1.33)—had a greater propensity toward favorable eHealth usage outcomes in internal practices and practices with patients. European general practitioners who positively valued information and communication technology (ICT) impact on their personal working processes (internal practices OR 5.30, 95% CI 4.73-5.93; practices with patients OR 4.83, 95% CI 4.32-5.40), teamwork processes (internal practices OR 4.19, 95% CI 3.78-4.65; practices with patients OR 3.38, 95% CI 3.05-3.74), and the doctor-patient relationship (internal practices OR 3.97, 95% CI 3.60-4.37; practices with patients OR 6.02, 95% CI 5.43-6.67) had a high propensity toward favorable effects of eHealth usage on internal practices and practices with patients. More favorable eHealth outcomes were also observed for self-employed European general practitioners (internal practices OR 1.33, 95% CI 1.22-1.45; practices with patients OR 1.10, 95% CI 1.03-1.28). Finally, general practitioners who reported that the number of patients treated in the last 2 years had remained constant (internal practices OR 1.08, 95% CI 1.01-1.17) or increased (practices with patients OR 1.12, 95% CI 1.03-1.22) had a higher propensity toward favorable eHealth usage outcomes.

**Conclusions:**

We provide new evidence of predictors (sociodemographic issues, attitudes toward ICT impacts, and working conditions) that explain favorable eHealth usage outcomes. The results highlight the need to develop more specific policies for eHealth usage to address different realities.

## Introduction

In recent years, advances in health information technologies (HITs), electronic health records (EHRs), personal health records (PHRs), electronic health (eHealth) and telehealth applications and devices, and health information exchange (HIE) systems have profoundly transformed professionals’ health care practice, thus, contributing to the efficiency of their activities by reducing errors, improving early diagnosis of diseases, and offering better management of chronic diseases [1-3]. In the European Union, these HIT and eHealth applications have been incorporated into hospitals and municipalities over the past 10 years, and a higher level of information integration and exchange has been achieved to the benefit of coordination and collaboration between and among health care professionals in all sectors [1,4,5]. In primary care, where 69% of general practitioners use internet-connected computers [1], EHR network usage is well established and widespread [6], whereas HIE system usage is less so [7]*.* In addition, growing emphasis is being placed on eHealth services as a way of enabling patients to have access to their medical information through PHRs [5,6].

The application of eHealth services to primary care is of particular interest because it is at this level of care where patients come into regular contact with health care systems. Primary care also provides the highest volume of health services [8]. Compared to current practice, however, general practitioners often consider eHealth services as being disruptive rather than supportive [7]. General practitioners are quite skeptical about the potential benefits of eHealth usage to citizens and patients [1] despite the perceived benefits [9]. Moreover, even though the data gathered by different health devices [10] and the Internet of Things [11,12] can serve as a complement to PHRs and help to identify patients’ health risks [11], the success of PHRs will be dependent on general practitioners’ attitudes and actions. Additional workload coupled with the lack of time, remuneration, information and communication technology (ICT) skills, interoperability, confidentiality, and clear rules about their liability stand out as the main barriers to eHealth usage alongside organizational issues and resistance to change [1,13-17].

While there is considerable evidence in the literature about the predictors of some particular uses of eHealth, attention has recently been drawn to the need to use more advanced methods and models to evaluate the shared health care model that eHealth proposes [18-21]. The construction of a new theoretical framework, a composite indicator of eHealth usage (by patients and health care professionals) that would allow us to understand the integrated and intersectoral workflow and dynamics of eHealth between health care professionals and patients within health care systems would be very useful. Thus, the public policies and strategic actions resulting from the research could be adapted more precisely to specific eHealth uses and the profiles of professionals or health care systems.

Earlier studies have already attempted to model and predict eHealth usage among patients in Europe [22]. The main aim of this work was to model and predict European general practitioners’ eHealth usage outcomes. We designed and tested a multidimensional model for this purpose. The results obtained provide new evidence of and have implications for the design of health organizations and public health policies.

## Methods

### Participants and Procedure

Data for this study were drawn from the Benchmarking Deployment of eHealth among General Practitioners Phase 2 (GPII) research project [23]. The survey was funded by the European Commission Directorate General for Communications Networks, Content and Technology. The GPII panel survey’s analysis had two main objectives: (1) measuring the level eHealth availability and usage in primary care and (2) examining what drives or hampers the overall level of eHealth adoption.

The study used survey data from a sample of 9196 European general practitioners, that is to say, physicians working in outpatient establishments in specialties such as general practice, family medicine, internal medicine, or general medicine. The sampling universe comprised 465,718 European physicians with an overall margin of error of ±1.03 in the case of maximum indetermination p=q=50%, for a confidence level of 95.5% (Multimedia Appendix 1). The sample had two essential characteristics. First, the survey selected a large sample of countries (31 in total): 27 European Union countries (Austria, Belgium, Bulgaria, Cyprus, Czech Republic, Denmark, Estonia, Finland, France, Germany, Greece, Hungary, Ireland, Italy, Latvia, Lithuania, Luxembourg, Malta, The Netherlands, Poland, Portugal, Romania, Slovakia, Slovenia, Spain, Sweden, and United Kingdom) plus Croatia, Iceland, Norway, and Turkey. Second, it selected an unequal-sized sample with country-specific margins of error varying between +4.15% and +13.84%. The margins of error for most of the countries in the sample (n=20) were around ±5.0%, whereas for 6 countries they were between ±6.0% and ±7.0%, and for only 5 countries (the smaller ones), they were above ±10.0%.

The questionnaire used in the survey contained 38 questions grouped into 3 dimensions (Multimedia Appendix 2). Part A covered general practitioners’ sociodemographic circumstances, organizational settings, practice location, and description of tasks and workload (10 questions). Part B covered the deployment and usage of ICT systems and functionalities (23 questions) and represented the core of the survey. After a set of general questions (basic infrastructure, interconnection with other system players, and security items), the following 4 pillars of eHealth usage measurement were addressed in this dimension: (1) EHRs, referring to the systems that are used by health care professionals to enter, store, view, and manage patient health and administrative information and data; (2) HIEs, referring to the process of electronically transferring, sharing, or enabling access to patient health information and data; (3) telehealth, covering the use of broadband-based technological platforms for the purpose of providing health services, medical training, and health education at a distance; and (4) PHRs, referring to the electronic systems allowing patients to have secure access to and manage their health information. Finally, Part C focuses on attitudes toward, perceived barriers to, and impacts of eHealth usage (5 questions).

The survey was answered by European general practitioners in mixed Web-based, phone (Web-CATI), and face-to-face interviews lasting for half an hour each and in a native language of each country. A study presentation paragraph was written to inform potential respondents about the confidentiality of any data provided and the academic aim of the research. European general practitioners voluntarily answered the questionnaire and did not receive any payment in cash or kind. While the questionnaire was being implemented, an expert was on hand at all times (via email) to resolve any queries that the respondents had. The respondent general practitioners were selected by means of probability sampling applied to each country universe. The net response rate was 35.5%. The fieldwork period ran from October 25, 2012 to March 6, 2013. The GPII research project followed the Checklist for Reporting Results of Internet E-Surveys criteria [24]. For a more detailed explanation, see the GPII research report [23].

### Data Analysis and Models

From an empirical perspective, explanatory factors determining eHealth usage outcomes raise two particular difficulties. First, the approach to the concept requires a multidimensional basis that is not usually captured in a single variable. In fact, the most common approaches found in the literature perform partial analyses of its various dimensions. This type of analysis has the disadvantage of not taking a full snapshot of the explanatory factors, which gives rise to the second difficulty: statistical modeling. In other words, eHealth usage outcomes can be interpreted as a latent, nonobservable concept, which, therefore, calls for statistical techniques that allow variables of this type, which are not directly measurable, to be used [22].

In the empirical literature, structural equation modeling (SEM) with latent variables has been used to overcome this problem. A general SEM is a formal mathematical model. It is a set of linear equations that encompasses various types of models such as regression analysis models, simultaneous equation systems, factor analysis, and path analysis. The main advantage of this method of analysis is the incorporation of different types of variables into the SEM. Directly observable and measurable variables, and theoretical or latent variables representing concepts that are not directly observed can, therefore, be incorporated. When the variable to be explained (dependent) is latent, it must be continuous, whereas dependent observed variables can be continuous, censored, binary, ordered, categorical (ordinals), or combinations of any of these variable types [25].

This method of analysis allows us to define eHealth usage outcomes as a latent variable, thus enabling us to calculate the specific explanatory effect of the variables that it comprises. Hence, besides building an overall explanatory model of the determinants of eHealth usage, it is also possible to identify which of its explanatory dimensions are more important. In addition, SEM enables the relationships between the different observable variables included in the model (indirect effects) to be estimated. In this context, and in order to capture the factors that explain eHealth usage outcomes in a large sample of European general practitioners, we proposed and tested a two-stage SEM with latent variables and measurement errors for 2012-2013.

We applied the 2-stage empirical methodology as follows: in the first stage, we tested the relationships among 101 indicators and the 9 dimensions describing eHealth usage and eHealth usage outcomes by means of SEM and additive indicators (in those dimensions with primary data of a dichotomous nature), and in the second stage, we tested the relationships among the indicators constructed for those 9 dimensions (based on the coefficients and aggregations from the first stage). This methodology involved the design and statistical testing of 5 empirical SEM models (4 models for the first stage and 1 model for the second stage) and also 5 additive indicators in the first stage.

The 9 model dimensions and variables are as follows:

- *Dimension 1: ICT usage (ICTUS)*, captured by a set of 5 variables measuring usage frequency (Multimedia Appendix 3);
- *Dimension 2: barriers to eHealth usage (BARRIERS)*, captured by a set of 16 variables measuring the factors that general practitioners regarded as barriers when evaluating eHealth usage (Multimedia Appendix 4);
- *Dimension 3: PHR usage (PHR)*, captured by a set of 6 variables measuring their usage or nonusage (Multimedia Appendix 5);
- *Dimension 4: telehealth (THEALTH)*, captured by a set of 4 variables measuring its usage or nonusage (Multimedia Appendix 6);
- *Dimension 5: HIE*, captured by a set of 15 variables measuring their usage or nonusage (Multimedia Appendix 7);
- *Dimension 6: Electronic Health Records_Decision Support Systems (EHR_DSS)*, captured by a set of 6 variables measuring their usage or nonusage (Multimedia Appendix 8);
- *Dimension 7*: *Electronic Health Records_Data (EHR_DAT)* captured by a set of 19 variables measuring their usage or nonusage (Multimedia Appendix 9);
- *Dimension 8: eHealth usage outcomes in internal practices (OUTINTPRA)*, captured by a set of 14 variables measuring the outcomes that general practitioners considered when evaluating eHealth usage in their internal practices (IP; Multimedia Appendix 10);
- *Dimension 9: eHealth usage outcomes in practices with patients (OUTPRAPAT)*, captured by a set of 16 variables measuring the outcomes that general practitioners considered when evaluating eHealth usage in their practices with patients (PP; Multimedia Appendix 11).

**Figure 1:**
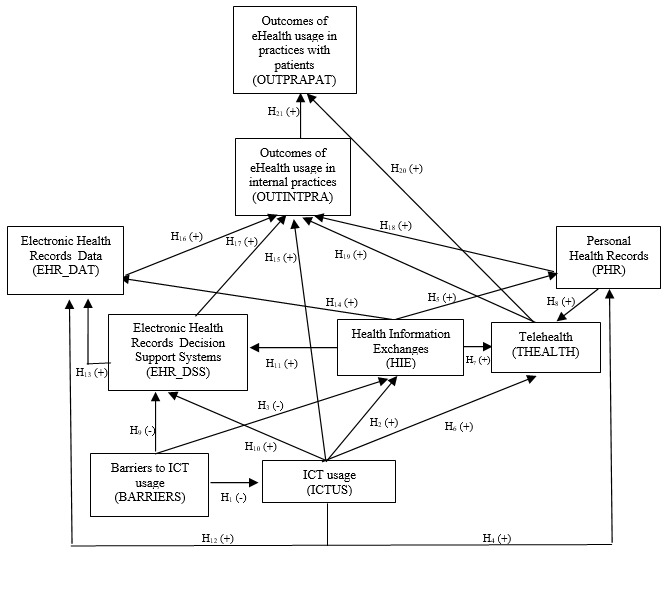
Model of eHealth usage and eHealth usage outcomes. H: hypothesis; ICT: information and communication technology; (+): positive prediction; (−) negative prediction.

Figure 1 shows the multidimensional model of eHealth usage dimensions and outcomes that we have tested.

Additionally, we performed logistic regression to model the predictors of 2 eHealth usage outcomes indicators using independent variables corresponding to sociodemographic circumstances, attitudes, and working conditions. For each independent variable, we calculated odds ratios (ORs) and their 95% CI. We used IBM SPSS Amos v24 (IBM Corp) for all calculations.

## Results

### eHealth Usage Outcomes

Multimedia Appendix 12 shows the results (standardized coefficients and measurement errors) of the first stage of estimating the explanatory factors of some dimensions of European general practitioners’ eHealth usage and of 2 dimensions of general practitioners’ eHealth usage outcomes for 2012-2013. In this first stage, we estimated the relationships among 51 indicators and 4 dimensions describing eHealth usage (ICTUS and BARRIERS) and eHealth usage outcomes (OUTINTPRA and OUTPRAPAT) using an SEM with measurement errors. First, it should be noted that all the variables specified in the model were statistically significant (99% confidence level). Second, the goodness-of-fit measurements for the 4 proposed models were highly satisfactory. Thus, the normed fit index (NFI), relative fit index (RFI), incremental fit index (IFI), Tucker-Lewis index (TLI), and comparative fit index (CFI) had very high values, approaching the optimal value of 1. The root mean square error of approximation (RMSEA) values were <0.08, thus, corroborating the validity of the estimated models.

In the ICTUS dimension, the standardized coefficient variability was 0.58. The variables with the highest explanatory power in this dimension were related to describing the medical organizations in contact with general practitioners (0.640) as well as the existence of problems of compatibility in electronically exchanging patient data (0.481). In contrast, fewer explanatory variables were related to computer usage in general practice (0.064). In the BARRIERS dimension, the standardized coefficient variability was much lower (0.15) between the explanatory variables related to the lack of time or additional workload (0.681), lack of sufficient training (0.673) or lack of sufficient ICT skills (0.663), and increased patient expectations (0.528).

Regarding the 2 dimensions of general practitioners’ eHealth usage outcomes, the standardized coefficients obtained showed a different variability. In the case of eHealth usage outcomes related to IP, the standardized coefficient variability was 0.37. The variables with the highest explanatory power were related to the fact that eHealth was easy to use (0.801), that general practitioners found it easy to get it to do what they wanted (0.769), and that it was flexible to use or interact with (0.744). The variables with the least explanatory power were related to people who influence general practitioners’ general behavior (0.442) or clinical behavior (0.435) in respect of eHealth usage. In the case of eHealth usage outcomes in PP, the standardized coefficient variability was much lower (0.15). The variables with the highest explanatory power were related to improvement in the efficiency of the whole health care system (0.797), in the quality of treatment (0.784), and in the quality of diagnosis decisions (0.783). In contrast, the variables with the least explanatory power were related to the general practitioners’ perceptions of eHealth usage increasing patient access to health care (0.647) or reducing pharmaceutical expenditure (0.649).

For the remaining 5 dimensions relating to eHealth usage, namely PHR, THEALTH, HIEs, EHR_DSS, and EHR_DAT, and as a result of the dichotomous nature of the base data, we constructed 5 additive indicators. For each of the 5 dimensions, we created a joint indicator that adds together their internal uses (indicators). Thus, the PHR indicator takes a value between 0 and 6, the THEALTH indicator between 0 and 4, the HIE indicator between 0 and 15, the EHR_DSS indicator between 0 and 6, and the EHR_DAT indicator between 0 and 19 (Multimedia Appendix 13). Some 37.58% (3456/9196) and 41.90% (3853/9196) of the general practitioners performed at least 1 of the 6 PHR practices and of the 4 THEALTH practices. However, the percentages in the HIE and EHR (DSS and DAT) dimensions were much higher. Some 87.99% (8092/9196), 70.20% (6456/9196), and 81.89% (7531/9196) of European general practitioners made at least 1 use of eHealth in the HIE and EHR (DSS and DAT) dimensions. After applying the coefficients—obtained from the first estimation stage—and the additive indicators, we constructed 7 composite indicators of eHealth usage and 2 of eHealth usage outcomes and determined their mean values (Table 1).

### eHealth Usage Outcomes in Internal Practices and in Practices With Patients

Table 2 shows the results (direct standardized coefficients and standard errors) of the second stage of modeling European general practitioners’ eHealth usage and eHealth usage outcomes for 2012-2013. In this second stage, we tested the relationships among the indicators constructed for the 7 dimensions describing eHealth usage (based on the coefficients and additive indicators from the first stage) and the 2 latent constructs of eHealth usage outcomes (based on the coefficients from the first stage) by using a 21-hypothesis SEM with standard errors (Figure 1). First, it should be noted that all the variables specified in the model were statistically significant (at least 95% confidence level). Second, the goodness-of-fit measurements for the proposed model were satisfactory. Thus, the NFI (0.966), RFI (0.914), IFI (0.967), TLI (0.915), and CFI (0.967) indices had high values, approaching the optimal value of 1. The RMSEA value was <0.08 (0.072), thus, corroborating the validity of the estimated model.

The direct standardized coefficients obtained validated the 21 formulated hypotheses and the signs of their relationships. For example, as we had hypothesized, the barriers to ICTUS determined a negative effect on ICT, HIE, and EHR_DSS usage. Regarding the relationships between different dimensions of eHealth usage, it is notable that ICTUS explained HIE (0.571) with considerable intensity that HIE had considerable explanatory power over PHR (0.398) and EHR_DSS (0.349) and that an important link was found between EHR_DSS and EHR_DAT (0.365). Concerning the indicators relating to eHealth usage outcomes, the indicator linked to IP was explained by ICT (0.132), EHR_DAT (0.215), EHR_DSS (0.050), PHR (0.112) and THEALTH (0.101) usage. In contrast, the outcomes indicator linked to PP was explained by THEALTH usage (0.027) and, primarily, by the IP outcomes indicator (0.607).

**Table 1:** Descriptive statistics of eHealth usage and eHealth usage outcome dimensions, 2012-2013.

#	Dimension	Mean (SD)	Minimum	Maximum	Skewness	Kurtosis
1	Information and communication technology usage	1.54 (1.002)	0.00	4.54	0.085	0.738
2	Barriers to eHealth usage	9.99 (3.014)	0.00	15.34	−0.855	1.177
3	Personal Health Records	0.82 (1.320)	0.00	6.00	1.872	3.360
4	Telehealth	0.62 (0.865)	0.00	4.00	1.450	1.747
5	Health Information Exchanges	5.26 (3.962)	0.00	15.00	0.551	−0.569
6	Electronic Health Records_Decision Support Systems	2.38 (2.127)	0.00	6.00	0.371	−1.233
7	Electronic Health Records_Data	13.80 (6.015)	0.00	19.00	−1.428	0.628
8	eHealth usage outcomes in internal practices	8.86 (2.719)	0.00	13.12	−0.785	0.681
9	eHealth usage outcomes in practices with patients	11.50 (3.712)	0.00	16.88	−0.785	0.741

**Table 2:** Explanatory factors of European general practitioners’ eHealth usage and eHealth usage outcomes, 2012-2013. Goodness-of-fit indices: normed fit index: 0.966; relative fit index: 0.914; incremental fit index: 0.967; Tucker-Lewis index: 0.915; comparative fit index: 0.967; root mean square error of approximation: 0.072.

Hypothesis (H)	Explained dimension	Explanatory dimension	Standardized coefficient^a^	*P* value of standardized coefficients	SE	*P* value of SEs
H1	ICTUS^b^	BARRIERS^c^	−0.118	<.001	0.003	<.001
H2	HIE^d^	ICTUS	0.571	<.001	0.034	<.001
H3	HIE	BARRIERS	−0.035	<.001	0.011	<.001
H4	PHR^e^	ICTUS	0.062	<.001	0.015	<.001
H5	PHR	HIEs	0.398	<.001	0.004	<.001
H6	THEALTH^f^	ICTUS	0.031	<.001	0.010	.003
H7	THEALTH	HIEs	0.248	<.001	0.003	<.001
H8	THEALTH	PHR	0.081	<.001	0.007	<.001
H9	EHR_DSS^g^	ICTUS	0.191	<.001	0.023	<.001
H10	EHR_DSS	BARRIERS	−0.012	<.001	0.006	.003
H11	EHR_DSS	HIEs	0.349	<.001	0.006	<.001
H12	EHR_DAT^h^	ICTUS	0.190	<.001	0.062	<.001
H13	EHR_DAT	EHR_DSS	0.365	<.001	0.027	<.001
H14	EHR_DAT	HIEs	0.174	<.001	0.016	<.001
H15	OUTINTPRA^i^	ICTUS	0.132	<.001	0.030	<.001
H16	OUTINTPRA	EHR_DAT	0.215	<.001	0.005	<.001
H17	OUTINTPRA	EHR_DSS	0.050	<.001	0.015	<.001
H18	OUTINTPRA	PHR	0.112	<.001	0.021	<.001
H19	OUTINTPRA	THEALTH	0.101	<.001	0.031	<.001
H20	OUTPRAPAT^j^	THEALTH	0.027	.002	0.036	.003
H21	OUTPRAPAT	OUTINTPRA	0.607	<.001	0.011	<.001

^a^Regression analysis: structural equation modeling (SEM); estimated coefficients: direct effects.

^b^ICTUS: information and communication technology usage.

^c^BARRIERS: barriers to information and communication technology usage.

^d^HIE: Health Information Exchange.

^e^PHR: Personal Health Records.

^f^THEALTH: telehealth.

^g^EHR_DSS: Electronic Health Records_Decision Support Systems.

^h^EHR_DAT: Electronic Health Records_Data.

^i^OUTINTPRA: eHealth usage outcomes in internal practices.

^j^OUTPRAPAT: eHealth usage outcomes in practices with patients.

### Predictors of eHealth Usage Outcomes in Internal Practices

To capture the main predictors of European general practitioners’ eHealth usage outcomes, we performed logistic regression using independent variables for general practitioners’ sociodemographic circumstances, attitudes toward ICT impact, and working conditions. The first step in this analysis was to recode the two composite indicators of eHealth usage outcomes. The mean value the composite indicator of OUTINTPRA was 8.86 (SD 2.72; minimum to maximum range 0.0-13.12). The mean value of the composite indicator of OUTPRAPAT was 11.5 (SD 3.71; minimum to maximum range 0.0-16.88). We, therefore, constructed a dichotomous eHealth usage outcomes indicator based on the mean of the composite indicators obtained. The two dichotomous eHealth usage outcomes indicators took the value 1 when the composite indicators of eHealth usage outcomes were equal to or greater than the mean and the value 0 when less than the mean. The mean value of the dichotomous composite indicator of OUTINTPRA was 0.547 (SD 0.498; minimum to maximum range 0-1). The mean value of the dichotomous indicator of OUTPRAPAT was 0.558 (SD 0.497; minimum to maximum range 0-1). For 54.75% (5035/9196) of European general practitioners, the eHealth usage outcomes in IP were more favorable (greater than the mean). For 55.82% (5133/9196) of European general practitioners, the eHealth usage outcomes in PP were more favorable (greater than the mean).

Table 3 shows the results of the logistic regression (OR) between the dichotomous composite indicator of OUTINTPRA and the independent variables. Regarding sociodemographic circumstances, European general practitioners who were female (OR 1.15, 95% CI 1.10-1.20) and younger (aged <35 years: OR 1.14, 95% CI 1.02-1.26 and aged 36-45 years: OR 1.16, 95% CI 1.06-1.28) had a higher propensity toward favorable OUTINTPRA than male general practitioners (OR 0.89, 95% CI 0.85-0.92) or those in older age groups (aged 46-55 years: OR 0.95, 95% CI 0.87-1.03 and aged >56 years: OR 0.90, 95% CI 0.82-0.98). We found no significant differences between European general practitioners’ workplace location and more favorable OUTINTPRA: large cities (>100,000 inhabitants): OR 1.02, 95% CI 0.93-1.11, small-midsized cities (20,000-100,000 inhabitants): (OR 0.98, 95% CI 0.89-1.07), and rural towns (<20,000 inhabitants): OR 1.00, 95% CI 0.97-1.03).

Concerning attitudes toward perceived ICT impact on IP, the results obtained showed considerable predictive power. European general practitioners who positively valued ICT impact on their personal working processes (OR 5.30, 95% CI 4.73-5.93), teamwork processes (OR 4.19, 95% CI 3.78-4.65), and the doctor-patient relationship (OR 3.97, 95% CI 3.60-4.37) had a high propensity toward favorable effects of OUTINTPRA. In contrast, European general practitioners who negatively valued the effect of ICT impact or considered that it had no effect on their personal working processes (no effect: OR 0.26, 95% CI 0.23-0.29; negatively: OR 0.12, 95% CI 0.09-0.15), teamwork processes (no effect: OR 0.31, 95% CI 0.28-0.35; negatively: OR 0.17, 95% CI 0.14-0.22), or the doctor-patient relationship (no effect: OR 0.51, 95% CI 0.47-0.56; negatively: OR 0.27, 95% CI 0.24-0.31) had a lower propensity toward favorable effects of eHealth usage. A clear link was also observed between a greater personal use of Web 2.0 (social media, blogs, etc) and more favorable OUTINTPRA. European general practitioners who had used 2.0 applications in their personal lives often (OR 1.77, 95% CI 1.60-1.97) or sometimes (OR 1.16, 95% CI 1.06-1.28) also showed a higher propensity toward favorable OUTINTPRA than those who rarely (OR 0.99, 95% CI 0.89-1.09) or never (OR 0.59, 95% CI 0.55-0.65) used 2.0 applications.

Finally, the study also found predictive power between some working conditions and favorable OUTINTPRA. Self-employed European general practitioners (OR 1.33, 95% CI 1.22-1.45) also had a greater predisposition toward favorable eHealth usage outcomes in IP than salaried European general practitioners (OR 0.82, 95% CI 0.75-0.89). For its part, the dynamic of the number of patients treated also had predictive power. In the case of favorable OUTINTPRA, the results obtained had an inverted U shape. European general practitioners who reported that the number of patients treated had remained constant in the last 2 years (OR 1.08, 95% CI 1.01-1.17) had a greater predisposition toward favorable eHealth usage outcomes in their IP. In contrast, general practitioners who reported that the number of patients treated had increased (1.03, 95% CI 0.95-1.12) or had fallen in recent years (OR 0.81, 95% CI 0.72-0.91) had less predictive power.

### Predictors of eHealth Usage Outcomes in Practices With Patients

Table 4 shows the results of the logistic regression (OR) between the dichotomous composite indicator of OUTPRAPAT and the independent variables. Regarding sociodemographic circumstances, European general practitioners who were female (OR 1.19, 95% CI 1.14-1.24) and younger (aged <35 years: OR 1.32, 95% CI 1.13-1.54 and aged 36-45 years: OR 1.21, 95% CI 1.10-1.33) had a higher propensity toward favorable OUTPRAPAT than male general practitioners (OR 0.86, 95% CI 0.83-0.89) or those in older age groups (aged 46-55 years: OR 0.84, 95% CI 0.77-0.92 and aged >56 years: OR 0.93, 95% CI 0.85-1.01). We found no significant differences between European general practitioners’ workplace location and more favorable OUTPRAPAT: large cities (>100,000 inhabitants): OR 0.97, 95% CI 0.89-1.05; small or midsized cities (20,000-100,000 inhabitants): OR 1.00, 95% CI 0.91-1.09; and rural towns (<20,000 inhabitants): OR 1.04, 95% CI 0.95-1.13.

Concerning attitudes toward perceived ICT impact on IP, the results obtained showed considerable predictive power. European general practitioners who positively valued ICT impact on their personal working processes (OR 4.83, 95% CI 4.32-5.40), teamwork processes (OR 3.38, 95% CI 3.05-3.74), and the doctor-patient relationship (OR 6.02, 95% CI 5.43-6.67) had a high propensity toward favorable effects of eHealth usage on PP. In contrast, European general practitioners who negatively valued the effect of ICT impact or considered that it had no effect on their personal working processes (no effect: OR 0.26, 95% CI 0.23-0.30; negatively: OR 0.17, 95% CI 0.13-0.21), teamwork processes (no effect: OR 0.38, 95% CI 0.34-0.42); negatively: OR 0.20, 95% CI 0.16-0.26), and the doctor-patient relationship (no effect: OR 0.38, 95% CI 0.35-0.42; negatively: OR 0.26, 95% CI 0.22-0.29) had a lower propensity toward favorable effects of eHealth usage. A clear link was also observed between a greater personal use of Web 2.0 (social media, blogs, etc) and more favorable OUTPRAPAT. European general practitioners who had used 2.0 applications in their personal lives often (OR 1.94, 95% CI 1.74-2.15) or sometimes (OR 1.19, 95% CI 1.08-1.31) also showed a higher propensity toward favorable OUTPRAPAT than those who rarely (OR 1.09, 95% CI 0.98-1.20) or never (OR 0.51, 95% CI 0.47-0.56) used 2.0 applications.

**Table 3:** Logistic regression (odds ratio, OR) models of the dichotomous composite indicator of eHealth usage outcomes in internal practices by sociodemographic circumstances, attitudes toward information and communication technology (ICT) impact, and working conditions, 2012-2013.

Sociodemographic factors		OR	95% CI
**Gender**			
	Male	0.89	0.85-0.92
	Female	1.15	1.10-1.20
**Age range (years)**			
	<35	1.14	1.02-1.26
	36-45	1.16	1.06-1.28
	46-55	0.95	0.87-1.03
	>56	0.90	0.82-0.98
**Workplace location**			
	Large city (more than 100,000 inhabitants)	1.02	0.93-1.11
	Small or midsized city (between 20,000 and 100,000 inhabitants)	0.98	0.89-1.07
	Rural town (fewer than 20,000 inhabitants)	1.00	0.97-1.03
**ICT impact on personal working processes**			
	Positive	5.30	4.73-5.93
	No change	0.26	0.23-0.29
	Negative	0.12	0.09-0.15
**ICT impact on teamwork processes**			
	Positive	4.19	3.78-4.65
	No change	0.31	0.28-0.35
	Negative	0.17	0.14-0.22
**ICT impact on the doctor-patient relationship**			
	Positive	3.97	3.60-4.37
	No change	0.51	0.47-0.56
	Negative	0.27	0.24-0.31
**Web 2.0 (social media, blogs, etc) usage in personal life**			
	Often	1.77	1.60-1.97
	Sometimes	1.16	1.06-1.28
	Rarely	0.99	0.89-1.09
	Never	0.59	0.55-0.65
**Occupational status**			
	Salaried	0.82	0.75-0.89
	Self-employed	1.33	1.22-1.45
**Number of patients treated in the last 2 years**			
	Increased	1.03	0.95-1.12
	Remained constant	1.08	1.01-1.17
	Decreased	0.81	0.72-0.91

**Table 4:** Logistic regression (odds ratio, OR) models of the dichotomous composite indicator of eHealth usage outcomes in practices with patients by sociodemographic circumstances, attitudes toward information and communication technology (ICT), and working conditions, 2012-2013.

Sociodemographic factors		OR	95% CI
**Gender**			
	Male	0.86	0.83-0.89
	Female	1.19	1.14-1.24
**Age range (years)**			
	<35	1.32	1.13-1.54
	36-45	1.21	1.10-1.33
	46-55	0.84	0.77-0.92
	>56	0.93	0.85-1.01
**Workplace location**			
	Large city (>100,000 inhabitants)	0.97	0.89-1.05
	Small or midsized city (20,000-100,000 inhabitants)	1.00	0.91-1.09
	Rural town (<20,000 inhabitants)	1.04	0.95-1.13
**ICT impact on personal working processes**			
	Positive	4.83	4.32-5.40
	No change	0.26	0.23-0.30
	Negative	0.17	0.13-0.21
**ICT impact on teamwork processes**			
	Positive	3.38	3.05-3.74
	No change	0.38	0.34-0.42
	Negative	0.20	0.16-0.26
**ICT impact on the doctor-patient relationship**			
	Positive	6.02	5.43-6.67
	No change	0.38	0.35-0.42
	Negative	0.26	0.22-0.29
**Web 2.0 (social media, blogs, etc) usage in personal life**			
	Often	1.94	1.74-2.15
	Sometimes	1.19	1.08-1.31
	Rarely	1.09	0.98-1.20
	Never	0.51	0.47-0.56
**Occupational status**			
	Salaried	1.02	0.93-1.11
	Self-employed	1.10	1.03-1.28
**Number of patients treated in the last 2 years**			
	Increased	1.12	1.03-1.22
	Remained constant	0.93	0.85-1.01
	Decreased	0.91	0.81-1.03

Finally, the study also found predictive power between some working conditions and favorable OUTPRAPAT. Self-employed European general practitioners (OR 1.10, 95% CI 1.03-1.28) also had a greater predisposition toward favorable OUTPRAPAT than salaried European general practitioners (OR 1.02, 95% CI 0.93-1.11). For its part, the dynamic of the number of patients treated also had predictive power. In the case of favorable OUTPRAPAT, the results obtained had a growing evolution. European general practitioners who reported that the number of patients treated had remained constant in the last 2 years (OR 1.12, 95% CI 1.03-1.22) had a greater predisposition toward favorable OUTPRAPAT. In contrast, general practitioners who reported that the number of patients treated had remained constant (OR 0.93, 95% CI 0.85-1.01) or had fallen (OR 0.91, 95% CI 0.81-1.03) did not have predictive power.

## Discussion

### Principal Findings

The goal of our study was to design and evaluate a predictive multidimensional model of general practitioners’ eHealth usage outcomes in IP and in PP, comprising 9 dimensions and 101 indicators. To that end, we used a broad population sample of 9196 European general practitioners. The results obtained are very useful for two reasons. First, obtaining new evidence centered solely on general practitioners allowed us to focus the analysis better, particularly with regard to the eHealth usage dimensions (ICTUS, BARRIERS, PHRs, HIEs, THEALTH, and EHRs) that determine favorable eHealth usage outcomes. Second, the predictors we obtained (sociodemographic circumstances, attitudes toward ICT impact, and working conditions) provided evidence that complements studies that have taken partial approaches.

### eHealth Usage Outcomes Indicators

In our study, we constructed 2 composite indicators using a 2-stage SEM methodology, and the results obtained are consistent with this evidence: they showed that in 2012-2013: (1) for 54.75% (5035/9196) of European general practitioners, the eHealth usage outcomes in IP were more favorable (greater than the mean) and (2) for 55.82% (5133/9196) of European general practitioners, the eHealth usage outcomes in PP were more favorable (greater than the mean).

The outcomes indicator linked to IP were explained by ICTUS, EHR_DAT, EHR_DSS, PHRs, and THEALTH. In contrast, the outcomes indicator linked to PP were explained by THEALTH usage and, primarily, by the IP outcomes indicator. General practitioners’ eHealth usage outcomes had a 2-fold interrelated dynamic. Firstly, the set of eHealth uses had explanatory power over IP outcomes. And secondly, the IP outcomes, together with THEALTH usage, determined eHealth usage outcomes in PP. In the explanation of general practitioners’ eHealth usage, it, therefore, seems that a certain “experience effect” occurs. eHealth usage takes place initially in IP and is then transferred to PP. In addition, the results of this study revealed the key role that telemedicine would play in the sense that, through training programs, upskilling, and learning, it would enable eHealth usage to be transferred from IP to PP [26].

Our study confirms that the perception of eHealth being easy to use, of general practitioners finding it easy to get it to do want they want, and of it being flexible to use or interact with are explanatory factors that have a bigger effect on eHealth usage in IP. The statistical significance of these determining factors refers back to the importance of perceived usefulness and ease of use when the use of a technology needs to be explained. General practitioners’ surgeries are characterized by the high number of patients cared for. Patients have various health problems, some of which are clinically complex. As a result, general practitioners have to handle several aspects at the same time, which may lead to doubts in their daily clinical practice [27]. That is why they need systems that are easy and flexible to use as well as being useful [28]. Hence the addition of “flexibility” to these two terms. The need to optimize time within a context of cutbacks in health care provision and spending may explain the significance of these determinants relating to general practitioners’ internal activities. Thus, if technology is adapted to the local context, does not entail any added complexity [29], and facilitates real-time access to data that is both reliable and fast, it will have an influence on its acceptance and adoption [28].

Regarding eHealth usage outcomes in PP, the variables with the highest explanatory power were related to improvement in the efficiency of the whole health care system, in the quality of treatment (0.784) and in the quality of diagnosis decisions. In this respect, eHealth is perceived as a technology that serves to reduce costs and increase the quality of health care provision [30-33]. General practitioners’ positive attitudes toward ICTUS explained and increased HIE usage. HIE had important explanatory power over PHR and EHR_DSS, and an important link between EHR_DSS and EHR_DAT was also found. Experiences that draw on the advantages of HIE and telemedicine to improve communication between general practitioners and their colleagues or specialists have been shown to be beneficial in terms of efficiency, cost-effectiveness, and improved medical care, with a high degree of satisfaction [32,34,35]. General practitioners’ adoption of EHR_DAT is crucial to compiling information across the entire health care system because they are the first point of contact in the provision of integrated health care supported by the potential of eHealth [36,37]. In addition, PHRs enable them to deal with all the patient’s self-reported information within the limited amount of time allocated to a clinical visit [38].

The variables with less explanatory power were related to general practitioners’ perceptions of whether or not eHealth usage increases patient access to health care or reduces pharmaceutical expenditure. Again, the variables with less explanatory power were related to the belief that electronic systems would disrupt health care provision[30,39]. It is important to ensure that the potential benefits of new technologies are clear to see within the organization through mechanisms of continuing evaluation and feedback [40].

### Predictors of eHealth Usage Outcomes (Internal Practices and Practices With Patients)

General practitioner’s eHealth usage in IP and in PP has been shown to have significant relationships with the individual characteristics of general practitioners, such as gender and age. It has been described in the literature that demographic factors such as age, education, gender, nationality, and clinical experience can have an influence on health care professionals’ predisposition toward eHealth systems [8,41,42].

Concerning attitudes toward the perceived ICT impact on internal and external practices, our analysis revealed that European general practitioners who positively valued ICT impact on their personal working processes, teamwork processes, and the doctor-patient relationship (IP OR 3.97, 95% CI 3.60-4.37) had a high propensity toward favorable OUTINTPRA. A clear link was also observed between a greater personal use of Web 2.0 (social media, blogs, etc) and more favorable OUTINTPRA. Various studies have described organizational and individual barriers to the implementation of eHealth services. Recognizing and understanding what the barriers and facilitators are is ideal for devising strategies and interventions to improve the effective eHealth usage and to address the barriers to implementation [43].

Regarding occupational status, our study has also revealed that self-employed European general practitioners had a greater predisposition toward favorable OUTINTPRA and OUTPRAPAT than salaried European general practitioners. Specifically, self-employed general practitioners attached importance to the potential of eHealth to reduce costs [30,32], despite the fact that practices with a single general practitioner might come up against higher barriers (eg, the costs of buying and maintaining technology) and face difficulties in terms of securing access to other essential resources for the implementation and continued use of eHealth services (eg, information technology support and training time) [44,45].

Last, from the perspective of demand pressures on health care practices, European general practitioners who reported that the number of patients treated had remained constant in the last 2 years had a greater predisposition toward favorable eHealth usage outcomes in their IP. In contrast, general practitioners who reported that the number of patients treated had increased or had fallen had less predictive power. This would, therefore, confirm the available evidence, which associates the implementation of digital technologies in the health care field with specific organizational circumstances, in particular with workflow pressures that are not too heavy [39,46,47].

In contrast—and this is the only result that clearly distinguishes between eHealth usage for IP and that for PP—demand pressures would have predictive power over positive eHealth outcomes. European general practitioners who reported that the number of patients treated had remained constant in the last 2 years had a greater predisposition toward favorable OUTPRAPAT [30,33,47]. While positive eHealth usage in IP was associated with demand that remained constant, in PP it would be associated with growing demand. Once again, it seems that the training effect prevails in the sense that eHealth usage is tested initially in IP, without the pressure of greater attention, and is then transferred to health care PP that are more pressurized.

### Limitations

Our study has several limitations. First, there was a time lag between the years the data were obtained (2012-2013) and the year we wrote the paper. However, we felt that the availability of a population database of 9196 European general practitioners deserved an analysis despite the time lag. In future research, and as they become available, we will use newer data and introduce dynamic comparisons. Second, the study provides information only from the perspective of physicians. In the future, we intend to address the issue of eHealth usage by health professionals and health users. By doing so, we will be able to improve our multidimensional approach and obtain mixing results and conclusions for all actors involved in eHealth usage and eHealth usage outcomes. Third, the empirical methodology could also be improved by looking at the intensity of eHealth usage (not simply usage or mean usage) and at a higher number of predictors.

### Conclusions

The results obtained highlight the need for more in-depth research to be conducted into the link between eHealth usage, eHealth outcomes and predictors, and the different health care systems in Europe. By doing so, it will be possible to increase the resolution of our results and to establish whether the intensity of eHealth usage and eHealth outcomes varies depending on the health care systems or the extent to which health care systems determine the prediction of eHealth usage or eHealth outcomes. Similarly, strategic and public policy actions resulting from the research could be adapted more precisely to each health care system. Finally, the study results could be supplemented by the construction of a composite indicator of eHealth usage by health care professionals and health care users. The design, validation, and prediction of composite indicators of eHealth usage and eHealth outcomes that take into consideration the perspectives of both users (ie, patients) and professionals in the different European health care systems would provide us with a very comprehensive view of the issue and would allow us to round off our multidimensional approach. We shall focus our efforts on all of these approaches in the near future.

Again, appropriate innovations are needed to promote eHealth usage. European, national and regional authorities should take the results of these studies into account to develop suitable policies for greater integration of HIT among European health care professionals. This setting poses significant challenges for the formulation of public policies and strategies by states where decisions about eHealth should not be overlooked.

## Appendix

Multimedia Appendix 1 Statistical information based on General Practitioners II (GPII) survey.

Multimedia Appendix 2 Benchmarking Deployment of eHealth among General Practitioners II (GPII) Questionnaire.

Multimedia Appendix 3 Information and communication technology (ICT) usage by European general practitioners descriptive statistics 2012-2013.

Multimedia Appendix 4 Barriers to eHealth usage by European general practitioners descriptive statistics 2012-2013.

Multimedia Appendix 5 Personal Health Records (PHR) usage by European general practitioners descriptive statistics 2012-2013.

Multimedia Appendix 6 Telehealth usage by European general practitioners descriptive statistics 2012-2013.

Multimedia Appendix 7 Health Information Exchanges (HIE) usage by European general practitioners descriptive statistics 2012-2013.

Multimedia Appendix 8 Electronic Health Records_ Decisions Support System (EHR_DSS) usage by European general practitioners descriptive statistics 2012-2013.

Multimedia Appendix 9 Electronic Health Records_ Data (EHR_DAT) usage by European general practitioners descriptive statistics 2012-2013.

Multimedia Appendix 10 Outcomes of internal practices in eHealth usage by European general practitioners descriptive statistics 2012-2013.

Multimedia Appendix 11 Outcomes of practices with patients in eHealth usage by European general practitioners descriptive statistics 2012-2013.

Multimedia Appendix 12 Explanatory factors of some dimensions of European general practitioners’ eHealth usage and eHealth usage outcomes in 2012-2013.

Multimedia Appendix 13 eHealth usage by European general practitioners dimensions frequency statistics* 2012-2013.

## Abbreviations

BARRIERS: barriers to eHealth usage
CFI: comparative fit index
eHealth: electronic health
EHR: electronic health record
EHR_DSS: electronic health records_decision support system
EHR_DAT: electronic health records_data
GPII: Benchmarking Deployment of eHealth among General Practitioners Phase 2
HIE: health information exchange
HIT: health information technology
ICT: information and communication technology
ICTUS: information and communication technology usage
IFI: incremental fit index
IP: internal practices
NFI: normed fit index
OR: odds ratio
OUTINTPRA: eHealth usage outcomes in internal practices
OUTPRAPAT: eHealth usage outcomes in practices with patients
PHR: personal health record
PP: practices with patients
RFI: relative fit index
RMSEA: root mean square error of approximation
SEM: structural equation modeling
THEALTH: telehealth
TLI: Tucker-Lewis index

## References

[1] Dinesen B, Nonnecke B, Lindeman D, Toft E, Kidholm K, Jethwani K, Young Heather M, Spindler Helle, Oestergaard Claus Ugilt, Southard Jeffrey A, Gutierrez Mario, Anderson Nick, Albert Nancy M, Han Jay J, Nesbitt Thomas (2016). Personalized Telehealth in the Future: A Global Research Agenda. J Med Internet Res.

[2] (2012). European Commission.

[3] Kuperman Gilad J (2011). Health-information exchange: why are we doing it, and what are we doing?. J Am Med Inform Assoc.

[4] Roehrs A, da Costa Cristiano André, Righi RDR, de Oliveira Kleinner Silva Farias (2017). Personal Health Records: A Systematic Literature Review. J Med Internet Res.

[5] (2014). European Commission.

[6] Huygens M, Vermeulen J, Friele R, van Schayck Onno Cp, de Jong Judith D, de Witte Luc P (2015). Internet Services for Communicating With the General Practice: Barely Noticed and Used by Patients. Interact J Med Res.

[7] Peeters J, Krijgsman J, Brabers A, Jong JD, Friele R (2016). Use and Uptake of eHealth in General Practice: A Cross-Sectional Survey and Focus Group Study Among Health Care Users and General Practitioners. JMIR Med Inform.

[8] Kidholm K, Stafylas P, Kotzeva A, Duedal Pedersen C, Dafoulas G, Scharf I, Kvistgaard Jensen L, Lindberg I, Stærdahl Andersen A, Lange M, Aletras V, Fasterholdt I, Stübin M, d’Angelantonio M, Ribu L, Grøttland A, Greuèl M, Isaksson L, Orsama A-L, Karhula T, Mancin S, Scavini C, Dyrvig A-K, Wanscher CE, Giannaokopoulos (2014). REgioNs of Europe WorkINg toGether for HEALTH Final Report - Public.

[9] Kidholm K, Stafylas P, Kotzeva A, Duedal Pedersen C, Dafoulas G, Scharf I (2014). REgioNs of Europe WorkINg toGether for HEALTH Final Report - Public.

[10] Riazul Islam SM, Humaun Kabir M, Hossain M, Daehan Kwak, Kyung-Sup Kwak (2015). The Internet of Things for Health Care: A Comprehensive Survey. IEEE Access.

[11] Gubbi J, Buyya R, Marusic S, Palaniswami M (2013). Internet of Things (IoT): A vision, architectural elements, and future directions. Future Generation Computer Systems.

[12] Li S, Xu Ld, Zhao S (2014). The internet of things: a survey. Inf Syst Front.

[13] Jones M, Koziel C, Larsen D, Berry P, Kubatka-Willms E (2017). Progress in the Enhanced Use of Electronic Medical Records: Data From the Ontario Experience. JMIR Med Inform.

[14] Ghazisaeedi M, Mohammadzadeh N, Safdari R (2014). Electronic Health Record (EHR) As a Vehicle for Successful Health Care Best Practice. Med Arch.

[15] Green LA, Potworowski G, Day A, May-Gentile R, Vibbert D, Maki B, Kiesel L (2015). Sustaining. Ann Fam Med.

[16] Shea C, Malone R, Weinberger M, Reiter K, Thornhill J, Lord J, Nguyen Nicholas G, Weiner Bryan J (2014). Assessing organizational capacity for achieving meaningful use of electronic health records. Health Care Manage Rev.

[17] Kruse C, Argueta D, Lopez L, Nair A (2015). Patient and provider attitudes toward the use of patient portals for the management of chronic disease: a systematic review. J Med Internet Res.

[18] Hogan T, Luger T, Volkman J, Rocheleau M, Mueller N, Barker A, Nazi Kim M, Houston Thomas K, Bokhour Barbara G (2018). Patient Centeredness in Electronic Communication: Evaluation of Patient-to-Health Care Team Secure Messaging. J Med Internet Res.

[19] Ammenwerth E, Schnell-Inderst P, Hoerbst A (2012). The impact of electronic patient portals on patient care: a systematic review of controlled trials. J Med Internet Res.

[20] Veroff D, Marr A, Wennberg DE (2013). Enhanced support for shared decision making reduced costs of care for patients with preference-sensitive conditions. Health Aff (Millwood).

[21] Parsi K, Chambers C, Armstrong A (2012). Cost-effectiveness analysis of a patient-centered care model for management of psoriasis. J Am Acad Dermatol.

[22] Torrent-Sellens J, Díaz-Chao Ángel, Soler-Ramos I, Saigí-Rubió Francesc (2016). Modelling and Predicting eHealth Usage in Europe: A Multidimensional Approach From an Online Survey of 13,000 European Union Internet Users. J Med Internet Res.

[23] Codagnone C, Lupiañez-Villanueva F (2013). Benchmarking Deployment of eHealth among General Practitioners - Final Report. European Union.

[24] Eysenbach G (2004). Improving the quality of Web surveys: the Checklist for Reporting Results of Internet E-Surveys (CHERRIES). J Med Internet Res.

[25] Marcoulides G, Moustaki I (2012). Latent variable and latent structure models.

[26] Saigí-Rubió Francesc, Torrent-Sellens J, Jiménez-Zarco Ana (2014). Drivers of telemedicine use: comparative evidence from samples of Spanish, Colombian and Bolivian physicians. Implement Sci.

[27] Carey M, Noble N, Mansfield E, Waller A, Henskens F, Sanson-Fisher R (2015). The Role of eHealth in Optimizing Preventive Care in the Primary Care Setting. J Med Internet Res.

[28] Lacasta Tintorer David, Manresa Domínguez Josep Maria, Pujol-Rivera E, Flayeh Beneyto Souhel, Mundet Tuduri Xavier, Saigí-Rubió Francesc (2018). Keys to success of a community of clinical practice in primary care: a qualitative evaluation of the ECOPIH project. BMC Fam Pract.

[29] Gagnon M, Nsangou É-R, Payne-Gagnon J, Grenier S, Sicotte C (2014). Barriers and facilitators to implementing electronic prescription: a systematic review of user groups' perceptions. J Am Med Inform Assoc.

[30] Steele Gray Carolyn, Gill A, Khan A, Hans P, Kuluski K, Cott C (2016). The Electronic Patient Reported Outcome Tool: Testing Usability and Feasibility of a Mobile App and Portal to Support Care for Patients With Complex Chronic Disease and Disability in Primary Care Settings. JMIR Mhealth Uhealth.

[31] Granja C, Janssen W, Johansen M (2018). Factors Determining the Success and Failure of eHealth Interventions: Systematic Review of the Literature. J Med Internet Res.

[32] Bashshur R, Shannon G, Krupinski E, Grigsby J (2013). Sustaining and realizing the promise of telemedicine. Telemed J E Health.

[33] Praveen D, Patel A, Raghu A, Clifford G, Maulik P, Mohammad Abdul Ameer, Mogulluru Kishor, Tarassenko Lionel, MacMahon Stephen, Peiris David (2014). SMARTHealth India: Development and Field Evaluation of a Mobile Clinical Decision Support System for Cardiovascular Diseases in Rural India. JMIR Mhealth Uhealth.

[34] Saigi-Rubió F, Jiménez-Zarco A, Torrent-Sellens J (2016). Determinants of the intention to use telemedicine: evidence from primary care physicians. Int J Technol Assess Health Care.

[35] Díaz-Chao A, Torrent-Sellens J, Lacasta-Tintorer D, Saigí-Rubió F (2014). Improving Integrated Care: Modelling the performance of an online community of practice. Int J Integr Care Internet.

[36] (2004). Atun R.

[37] Macinko J, Starfield B, Shi L (2003). The Contribution of Primary Care Systems to Health Outcomes within Organization for Economic Cooperation and Development (OECD) Countries, 1970-1998. Health Serv Res.

[38] Ford E, Hesse B, Huerta T (2016). Personal Health Record Use in the United States: Forecasting Future Adoption Levels. J Med Internet Res.

[39] Janols R, Lind T, Göransson Bengt, Sandblad B (2014). Evaluation of user adoption during three module deployments of region-wide electronic patient record systems. Int J Med Inform.

[40] Ross J, Stevenson F, Lau R, Murray E (2016). Factors that influence the implementation of e-health: a systematic review of systematic reviews (an update). Implement Sci.

[41] Goldstein DH, Phelan R, Wilson R, Ross-White A, VanDenKerkhof EG, Penning JP, Jaeger M (2014). Brief review: Adoption of electronic medical records to enhance acute pain management. Can J Anaesth.

[42] Jamoom E, Patel V, Furukawa M, King J (2014). EHR adopters vs. non-adopters: Impacts of, barriers to, and federal initiatives for EHR adoption. Healthc (Amst).

[43] Ross J, Stevenson F, Lau R, Murray E (2015). Exploring the challenges of implementing e-health: a protocol for an update of a systematic review of reviews. BMJ Open.

[44] Nazi K (2013). The personal health record paradox: health care professionals' perspectives and the information ecology of personal health record systems in organizational and clinical settings. J Med Internet Res.

[45] Gagnon M, Desmartis M, Labrecque M, Car J, Pagliari C, Pluye P, Frémont Pierre, Gagnon Johanne, Tremblay Nadine, Légaré France (2012). Systematic review of factors influencing the adoption of information and communication technologies by healthcare professionals. J Med Syst.

[46] Embi P, Weir C, Efthimiadis E, Thielke S, Hedeen A, Hammond K (2013). Computerized provider documentation: findings and implications of a multisite study of clinicians and administrators. J Am Med Inform Assoc.

[47] Hao W, Hsu Y, Chen K, Li H, Iqbal U, Nguyen Phung-Anh, Huang Chih-Wei, Yang Hsuan-Chia, Lee Peisan, Li Mei-Hsuan, Hlatshwayo Sharoon Lungile, Li Yu-Chuan Jack, Jian Wen-Shan (2015). LabPush: a pilot study of providing remote clinics with laboratory results via short message service (SMS) in Swaziland, Africa - a qualitative study. Comput Methods Programs Biomed.

