# Supplementary material for: Modeling and Predicting Outcomes of eHealth Usage by European Physicians: Multidimensional Approach from a Survey of 9196 General Practitioners
Source: J Med Internet Res. 2018 Oct 22;20(10):e279. doi: 10.2196/jmir.9253 (PMC6231736; doi:10.2196/jmir.9253)
Supplement: Multimedia Appendix 1 [file jmir_v20i10e279_app1.pdf]

Appendix I. Statistical information based on General Practitioners II (GPII) survey

Population	General practitioners (physicians working in outpatient establishments in specialities such as general practice, family doctor, internal medicine or general medicine).
Scope of countries	27 EU countries (Austria, Belgium, Bulgaria, Cyprus, Czech Republic, Denmark, Estonia, Finland, France, Germany, Greece, Hungary, Ireland, Italy, Latvia, Lithuania, Luxembourg, Malta, Netherlands, Poland, Portugal, Romania, Slovakia, Slovenia, Spain, Sweden and United Kingdom) plus Croatia, Iceland, Norway and Turkey.
Type of survey	Mixed (Online, phone: Web-CATI, and face-to-face).
Sample size	9,196 interviews in total (N per country varies in a range between 50 and 572 general practitioners).
Sample extraction and weighting	Simple random sample and weighting by country to be able to interpret the overall data.
Sampling error	±1.03% for overall sample (31 countries). Sample error per country-specific samples varies in a range between ±4.15% and ±13.84%. In all cases, a maximum indeterminate probability (p=q=50), for a confidence level of 95.5% is applicable for each country.
Response rate	Varied between 30% and 40%.

Source: GPII.
