# Supplementary material for: Modeling and Predicting Outcomes of eHealth Usage by European Physicians: Multidimensional Approach from a Survey of 9196 General Practitioners
Source: J Med Internet Res. 2018 Oct 22;20(10):e279. doi: 10.2196/jmir.9253 (PMC6231736; doi:10.2196/jmir.9253)
Supplement: Multimedia Appendix 2 [file jmir_v20i10e279_app2.pdf]

Appendix 2. Benchmarking Deployment of eHealth among General Practitioners II (GPII) Questionnaire

The questions that follow will be about your work in relation to the use of Information and Communication Technologies (also referred to as "ICT"). The use of ICT in healthcare is also referred to as "eHealth". Due to different understanding of the concept of "eHealth" in different contexts, we will use the more generic expression "ICT", but please bear in mind that we refer to **Information and Communication Technologies explicitly devised to support the provision of healthcare.**

A. GPs socio-demographics, individual characteristics and tasks description

Q1. Gender:

Female
Male

Q2. Age:

____ years old [AGE SHOULD BE BETWEEN 23 AND 75 YEARS OLD; IF NOT, CLOSE]

Q3. Which of the following statements best reflects your professional status? [SINGLE ANSWER]

I am a salaried General Practitioner (GP) working in a Health Centre → ASK Q4
I am a self-employed GP working alone (with only administrative support staff) in my own practice → GO TO Q5
I am a self-employed GP working in a group practice with other physicians → ASK Q4
Other → ASK Q4

Q4. Approximately (estimate if you do not know exactly) how many physicians work at your health centre or private group practice, <u>including</u> yourself?

Number of GPs including yourself (or full-time equivalent): ____
Number of other physicians (or full-time equivalent): ____

Q5. Has the number of patients you treat decreased, remained stable, or increased over the past two (2) years? [SINGLE ANSWER]

Decreased
Remained stable
Increased
I don't know

Q6. Your work place is located in a…? [SINGLE ANSWER]

large city (more than 100,000 inhabitants)
mid-small city (between 20,000 to 100,000 inhabitants)
rural town (less than 20,000 inhabitants)

Q7. How many years have you spent in general practice?

____ years [GPs SHOULD HAVE AT LEAST 1 YEAR OF EXPERIENCE; IF NOT, CLOSE]

Q8. How many minutes per week do you have direct contact (either face-to-face or by phone) with…? [MULTIPLE ANSWER; MUST ANSWER AT LEAST 2 ITEMS]

1. Other General Practitioners approx. ______ minutes / week
2. Specialists approx. ______ minutes / week
3. Practice nurses approx. ______ minutes / week
4. Home care nurses approx. ______ minutes / week

5. Physiotherapists approx. ______ minutes / week
6. Social workers (including youth care) approx. ______ minutes / week

Q9. When your patient has been seen by a specialist or discharged by a hospital, how often does the following occur? [ONE ANSWER PER ITEM]

	Always	Often	Some-times	Rarely	Never
You receive a report back from the specialist/hospital with all relevant health information					
The information you receive is timely, that is, received in a timely manner so that it is available when needed					

Q10. How do you usually receive or access this information (if at all)?
[MULTIPLE ANSWER]

1. By fax
2. By mail or courier
3. By electronic mail (e-mail)
4. I have (electronic) remote access to it
5. The patient hands it to me
6. Other
7. I don’t receive/access it at all

B. Deployment and Usage of Information and Communication Technologies (ICT) Systems and Functionalities

Q11. Does your practice or department of general practice use computers? [SINGLE ANSWER]

1. Yes
2. No → EXPLAIN “DEPARTMENT” IF NECESSARY*; CLOSE & RECORD
3. I don’t know → EXPLAIN “DEPARTMENT” IF NECESSARY*; CLOSE & RECORD

*NOTE: “department of general practice” means the unit you work for (in a health centre, polyclinic, hospital, etc.)

Q12a. Do you have access to a computer in the consultation room? [SINGLE ANSWER]

1. Yes → ASK Q12b
2. No → GO TO Q12d
3. I don't know → GO TO Q12d

Q12b. Do you use the computer during consultations? [SINGLE ANSWER]

1. Yes → ASK Q12c
2. No → GO TO Q12d & SKIP Q12g
3. I don't know → GO TO Q12d & SKIP Q12g

Q12c. Do you use the computer to show patients any health-related information during consultations? [SINGLE ANSWER]

1. Yes, routinely
2. Yes, occasionally
3. No
4. I don't know

Q12d. Does your practice or department of general practice have access to the Internet? [SINGLE ANSWER]

1. Yes → ASK Q12e
2. No → GO TO Q13
3. I don’t know → GO TO Q13

Q12e. What type of connection to the Internet does your practice have? [MULTIPLE ANSWER IF VARIOUS CONNECTIONS]

1. Dial Modem
2. ISDN connection
3. DSL connection

4. Other broadband connection
5. Mobile Internet connection while on the move or outside the practice
6. I don't know

Q12f. **What speed does the connection reach?** [MULTIPLE ANSWER IF VARIOUS CONNECTIONS]

1. Less than or equal to 2 MBps (megabits per second)
2. Between 2 and 30 MBps (megabits per second)
3. Between 30 and 100 MBps (megabits per second)
4. More than 100 MBps (megabits per second)
5. I don’t know

Q12g. **Do you use the Internet during consultations?** [SINGLE ANSWER]

1. Yes, routinely
2. Yes, occasionally
3. No, I do not use it
4. No, I do not have access to the Internet in the consultation room
5. I don't know

Q13. **<u>When you are working outside your office (i.e. visiting a patient)</u>, which of the following devices do you have at your disposal?**
[FOR ITEMS ANSWERED AS “YES”] **Do you use them <u>when you are working outside your office</u>?**

	HAVE IT			USE IT		
	**Yes**	**No**	**I don’t know**	**Yes, routinely**	**Yes, occasionally**	**No, I don’t use it**
A simple mobile phone (with no internet connection)						
A mobile phone with internet connection and e-mail (called “smartphone”)						
A laptop with internet connection						
A laptop with no internet connection						
A tablet (e.g. iPad)						

Q14. **Does your office or health centre have its own website?** [SINGLE ANSWER]

Yes
No
I don’t know

Q15. **Do the computers and other ICT systems in use at your office have any of the following security features?** [MULTIPLE ANSWER]

Electronic signature
Encryption of sent or received files and e-mails
Password protection of sent or received files
Password-protected access
I don’t know

Q16. **How often do you encounter problems of compatibility when exchanging patient data electronically?** [SINGLE ANSWER]

Very often
Often

Sometimes
Seldom
I don’t exchange patient data
I don’t know

Q17. **To which of the following organisations or persons is your office’s ICT system connected electronically?**
[Note: “ICT” stands for “Information and Communication Technologies”]
[FOR ITEMS ANSWERED AS “YES”] **Do you use the connections to the following organisations/persons?**

	**CONNECTED**			**USE IT**		
	**Yes**	**No**	**I don’t know**	**Yes, routinely**	**Yes, occasionally**	**No, I don’t use this connection**
Other GPs						
Specialist practices						
Hospitals						
Laboratories						
Pharmacies						
Care homes						
Patients' homes						
Health authorities						
Insurance companies						
Suppliers						
Others						

Q18. **How are administrative and patient medical records stored at your office?**
[SINGLE ANSWER]

All electronic
Mostly electronic
Combined electronic/paper
Mostly paper
All paper

Q19a. **In your practice, do you record and store patients’ medical and administrative data electronically?**
[SINGLE ANSWER]

Yes → GO TO Q20a
No → ASK Q19b
I don’t know → GO TO Q20a

Q19b. [IF OFFICE HAS NO ELECTRONIC HEALTH RECORDS ACCORDING TO Q19a]
Why doesn’t your office have these systems? [MULTIPLE ANSWER]

Too expensive
Not needed
Not useful
Too complicated
Still unsure about privacy and confidentiality issues
Other

Q20a. Does your ICT system allow you to record and store the following types of patient data?

[Note: "ICT" stands for "Information and Communication Technologies"]

[FOR ITEMS ANSWERED AS "YES"] **Do you use them?**

	ALLOW IT			USE IT		
	**Yes**	**No**	**I don't know**	**Yes, routinely**	**Yes, occasionally**	**No, I don't use it**
Basic medical parameters (e.g. allergies)						
Vital signs						
Treatment outcomes						
Problem list / diagnoses						
Medication list						
Immunizations						
Medical history						
Patient demographics						
Lab test results						
Radiology test reports						
Radiology test images						
Symptoms (reported by patient)						
Reason for appointment						
Clinical notes						
Prescriptions / medications						
Ordered tests						
Create/update disease management/ care plan (e.g., diabetes)						
Finances / billing						
Administrative patient data						

Q20b. Does your ICT system have any of the clinical decision support functionalities listed below (such as real-time alerts or prompts)?

[Note: "ICT" stands for "Information and Communication Technologies"]

[FOR ITEMS ANSWERED AS "YES"] **Do you use them?**

	HAVE IT			USE IT		
	**Yes**	**No**	**I don't know**	**Yes, routinely**	**Yes, occasionally**	**No, I don't use it**
Clinical guidelines and best practices (e.g., alerts, prompts)						
Drug-drug interactions						
Drug-allergy alerts						
Drug-lab interactions						
Contraindications (e.g., based on age, gender, pregnancy status)						
Be alerted to a critical laboratory value						

Q21. Does your ICT system allow you to transfer/share/enable/access patient data electronically, permitting you to engage in any of the following?

[Note: "ICT" stands for "Information and Communication Technologies"]

[FOR ITEMS ANSWERED AS "YES"] **Do you use them?**

ALLOW IT			USE IT		
**Yes**	**No**	**I don't know**	**Yes, routinely**	**Yes, occasionally**	**No, I don't use it**

Interact with patients by email about health-related issues						
Patient appointment requests						
Make appointments at other care providers on your patients' behalf						
Send/receive referral and discharge letters						
Order supplies for your practice						
Transfer prescriptions to pharmacists						
Exchange medical patient data with other healthcare providers and professionals						
Receive laboratory reports						
Receive and send laboratory reports and share them with other healthcare professionals/providers						
Exchange patient medication lists with other healthcare professionals / providers						
Exchange radiology reports with other healthcare professionals / providers						
Exchange medical patient data with any healthcare provider in other countries						
Certify sick leaves						
Certify disabilities						
Exchange administrative patient data with reimbursers or other care providers						

Q22. **"Telehealth" is the use of broadband-based technological platforms for the purpose of providing health services, medical training and health education over a distance. Which of the following telehealth services do you currently have access to?**

[FOR ITEMS ANSWERED AS "YES"] **Do you use the following "telehealth" services?**

	ACCESS TO IT			USE IT		
	**Yes**	**No**	**I don't know**	**Yes, routinely**	**Yes, occasionally**	**No, I don't use it**
Training/Education						
Consultations with other healthcare practitioners						
Consultations with patients						
Monitoring patients remotely at their homes (i.e. "telemonitoring")						

Q23. [IF ANSWERED "YES" TO USAGE in Q22 "Monitoring patients remotely"]

You said that you provide telemonitoring service to patients at their homes. How is the service paid for? [MULTIPLE ANSWER]

I provide the service as part of my mandate and contract obligations (no additional payment / funding is required)

The service is fully reimbursed by the national health system

The service is fully reimbursed by social insurance fund

The service is provided only for patients with a private insurance coverage

The service is partially reimbursed (by national health system or social health insurance or private insurance) and partially paid by patients

The service is entirely paid by patients and is not reimbursed

Other
I don’t know

Q24a. Does your ICT system allow patients to have secure access to and manage their health information and/or other services such as referrals, appointments, etc.?

[Note: “ICT” stands for “Information and Communication Technologies”]

[SINGLE ANSWER]

Yes, they can both access and manage their information and data
Yes, they can view their information and data, but cannot manage it
No, neither access nor manage
I don’t know

Q24b. Does your ICT system give patients online access to the following services?

[Note: “ICT” stands for “Information and Communication Technologies”]

[FOR ITEMS ANSWERED AS “YES”] **Do your patients use these services?**

	**ALLOW IT**			**USE IT**		
	**Yes**	**No**	**I don’t know**	**Yes, routinely**	**Yes, occasionally**	**No, they don’t use it**
Request referrals						
Request appointments						
Request renewals or prescriptions						
View their medical records						
Supplement their medical records						
View test results						

C. Barriers, Impact, Attitudes

Q25. To what extent do you agree/disagree with the following statements related to the use of Information and Communications Technologies (ICT) in your practice?
[ONE ANSWER PER ITEM]

	Strongly agree	Somewhat agree	Somewhat disagree	Strongly disagree	I don't know
Useful for my practice					
Increases the number of patients I can see on average during working hours					
Enhances effectiveness of job					
Increases quality of care					
Easy to use					
Easy to get it to do what I want					
Flexible to use/interact with					
Colleagues who are important to me think I should use ICT systems					
People who influence my behaviour think I should use ICT systems					
People who influence my clinical behaviour think I should use ICT systems					
I have necessary resources to use ICT systems					
I have knowledge to use ICT systems					
I have technical assistance available					
Using ICT systems is entirely under my control					

Q26. To what extent do you agree/disagree that the following items are <u>barriers</u> to the introduction and usage of ICT systems in primary care?
[Note: "ICT" stands for "Information and Communication Technologies"]
[ONE ANSWER PER ITEM]

	Strongly agree	Somewhat agree	Somewhat disagree	Strongly disagree	I don't know
Lack of financial incentives					
Lack of financial resources					
Lack of access to the technology					
Lack of technical support					
Lack of inter-operability and standards					
Lack of sufficient resilience (ICT systems can fail)					
Lack of sufficient security and risk control					
Lack of framework (regulatory, legislative, ethical) on confidentiality and privacy issues					
Lack of time / additional workload					
Lack of sufficient ICT skills on the side of healthcare professionals					
Lack of sufficient training for healthcare professionals					

Lack of clear motivation to use ICT (not sure about its usefulness)					
Increased patients expectations					
Lack of framework on using e-mail between doctors and patients (i.e. standards for response time)					
Lack of remuneration for additional work answering patients' e-mails					
Difficult to use					

Q27. To what extent do you agree/disagree that the following items are <u>positive effects</u> from the introduction of ICT systems in primary care?

[Note: "ICT" stands for "Information and Communication Technologies"]

[ONE ANSWER PER ITEM]

	**Strongly agree**	**Somewhat agree**	**Somewhat disagree**	**Strongly disagree**	**I don't know**
Reduce medical errors					
Improvement in the quality of diagnosis decisions					
Improvement in the quality of treatment					
Enhance self-evaluation					
More data for clinical research and public health					
Facilitate patients' education and adherence to prescriptions					
Improvement in patients' satisfaction					
Increased patients' access to healthcare (i.e. booking online appointment, viewing their data)					
Avoid unnecessary tests and duplications					
Increase average number of patients receiving help during one day					
Reduce pharmaceutical expenditure					
Shorter waiting lists					
Allow more efficient consultations					
Improvement in coordination between the different levels of the health system					
Expedite workflow due to the availability of patients clinical data					
Improvement in the efficiency of the whole health system					

Q28. In what ways has the use of Information and Communication Technologies (ICT) systems changed your work practice? Has it had a positive influence, a negative influence, or no change at all on it?
[ONE ANSWER PER ITEM]

	Positive	No change	Negative	I don't know
Your personal working processes				
Your staff working processes				
Quality of diagnosis and treatment decisions				
Doctor-patient relationship				

Q29. Some patients use the Internet to search information about their symptoms or conditions. How often do you encounter one of the following situations concerning health-related information your patients found online?
[ONE ANSWER PER STATEMENT]

	Often	Sometimes	Rarely	Never
Patients wanted to discuss the information they found online during consultation				
Patients misapplied or misunderstood the information they found online				
The Information your patients found online was actually beneficial for them				
You recommended specific websites to your patients				
Chronically-ill patients told you that internet is helping them in the self-management of their illness				

Q30. Social networking sites, blogs, and other online tools are part of the so-called "Web 2.0". To what extent do you use any of these tools… ?
[ONE ANSWER PER STATEMENT]

	Often	Sometimes	Rarely	Never
in your practice with patients				
in your practice with other healthcare professionals				
in your private life				

CLOSURE

You have successfully completed the online survey. Thank you very much for your time. Your feedback is very important.

For quality control purposes, we would appreciate it if you could give us the following information about yourself. Please note this information will be deleted after the study is finished.

Full Name: ………………………………………………..

Phone Number: …………………………………………….

Region (please select your geographic area):

___ Area 1
___ Area 2
___ Area 3
___ Area 4
___ Area 5
