# Supplementary material for: Modeling and Predicting Outcomes of eHealth Usage by European Physicians: Multidimensional Approach from a Survey of 9196 General Practitioners
Source: J Med Internet Res. 2018 Oct 22;20(10):e279. doi: 10.2196/jmir.9253 (PMC6231736; doi:10.2196/jmir.9253)
Supplement: Multimedia Appendix 3 [file jmir_v20i10e279_app3.pdf]

Appendix 3a. ICT usage by European general practitioners descriptive statistics. 2012-2013

	N	Mean	Std. Dev.	Minimum	Maximum	Skewness	Kurtosis
1. Internet usage during consultations	9,196	1.34	0.686	0	2	-0.566	-0.779
2. Computers usage in general practice	9,196	0.99	0,052	0	1	-19.104	336.041
3. Computers usage to show patients health-related information	9,196	1.20	0.687	0	2	-0.275	-0.892
4. Problems of compatibility in electronically exchanging patient data	9,196	1.42	1.257	0	3	0.027	-1.654
5. Medical organizations in contact with general practitioner	9,196	2.69	2.192	0	11	0.897	0.652

Source: Own elaboration.

Appendix 3b. ICT usage by European general practitioners frequency statistics. 2012-2013

		Valid percentage											
	N	0	1	2	3	4	5	6	7	8	9	10	11
1. Internet usage during consultations*	9,196	12.3	41.1	46.7	-	-	-	-	-	-	-	-	-
2. Computers usage in general practice**	9,196	0.3	99.7	-	-	-	-	-	-	-	-	-	-
3. Computers usage to show patients health-related information***	9,196	15.7	48.9	35.4	-	-	-	-	-	-	-	-	-
4. Problems of compatibility in electronically exchanging patient data****	9,196	38.3	9.6	23.5	28.6	-	-	-	-	-	-	-	-
5. Medical organizations in contact with general practitioner*****	9,196	16.3	19.2	18.3	14.1	12.1	9.4	4.8	2.5	1.5	0.9	0.5	0.3

* 0=No, I do not use it or not availability; 1=Yes, occasionally; 2=Yes, routinely.

** 0=No; 1=Yes.

*** 0=No, I do not use it or not availability; 1=Yes, occasionally; 2=Yes, routinely.

**** 0=I don’t exchange patient data; 1=Often; 2=Sometimes; 3=Seldom.

***** Number of medical organizations in contact with general practitioner= from 0 to 11.

Source: Own elaboration.
