# Supplementary material for: Modeling and Predicting Outcomes of eHealth Usage by European Physicians: Multidimensional Approach from a Survey of 9196 General Practitioners
Source: J Med Internet Res. 2018 Oct 22;20(10):e279. doi: 10.2196/jmir.9253 (PMC6231736; doi:10.2196/jmir.9253)
Supplement: Multimedia Appendix 4 [file jmir_v20i10e279_app4.pdf]

Appendix 4a. Barriers to eHealth usage by European general practitioners descriptive statistics. 2012-2013

	N	Mean	Std. Dev.	Minimum	Maximum	Skewness	Kurtosis
6. Lack of financial incentives	9,196	2.81	1.180	0	4	-0.838	-0.201
7. Lack of financial resources	9,196	2.81	1.175	0	4	-0.846	-0.164
8. Lack of access to the technology	9,196	2.44	1.153	0	4	-0.331	-0.819
9. Lack of technical support	9,196	2.57	1.141	0	4	-0.519	-0.583
10. Lack of inter-operability and standards	9,196	2.61	1.303	0	4	-0.767	-0.518
11. Lack of sufficient resilience –ICT systems can fail-	9,196	2.63	1.175	0	4	-0.780	-0.186
12. Lack of sufficient security and risk control	9,196	2.54	1.195	0	4	-0.612	-0.490
13. Lack of framework on confidentiality and privacy issues	9,196	2.58	1.237	0	4	-0.625	-0.695
14. Lack of time –additional workload-	9,196	2.80	1.131	0	4	-0.730	-0.361
15. Lack of sufficient ICT skills by general practitioners	9,196	2.75	1.061	0	4	-0.787	0.054
16. Lack of sufficient training for health care professionals	9,196	2.82	1.052	0	4	-0.860	0.213
17. Lack of clear motivation to use ICT	9,196	2.47	1.143	0	4	-0.375	-0.791
18. Increased patients expectations	9,196	2.32	1.203	0	4	-0.380	-0.760
19. Lack of framework on using e-mail between doctors and patients	9,196	2.58	1.273	0	4	-0.721	-0.530
20. Lack of remuneration for additional work answering patients e-mails	9,196	2.92	1.236	0	4	-1.028	0.002
21. Difficult to use	9,196	2.20	1.079	0	4	-0.132	-0.732

Source: Own elaboration.

Appendix 4b. Barriers to eHealth usage by European general practitioners frequency statistics. 2012-2013

		Valid percentage*				
	N	0	1	2	3	4
6. Lack of financial incentives	9,196	5.8	10.3	15.8	33.9	34.3
7. Lack of financial resources	9,196	5.7	9.9	16.1	33.9	34.4
8. Lack of access to the technology	9,196	5.2	18.7	23.2	33.0	20.0
9. Lack of technical support	9,196	5.0	14.7	21.2	36.0	23.0
10. Lack of inter-operability and standards	9,196	11.8	8.9	14.8	35.7	28.8
11. Lack of sufficient resilience –ICT systems can fail-	9,196	7.9	9.4	18.4	40.1	24.3
12. Lack of sufficient security and risk control	9,196	8.1	11.6	21.2	35.9	23.1
13. Lack of framework on confidentiality and privacy issues	9,196	8.4	12.1	19.1	33.3	27.1
14. Lack of time –additional workload-	9,196	3.7	12.2	16.8	34.4	32.8
15. Lack of sufficient ICT skills by general practitioners	9,196	4.0	10.1	18.1	43.0	24.9
16. Lack of sufficient training for health care professionals	9,196	3.6	9.0	16.8	42.4	28.1
17. Lack of clear motivation to use ICT	9,196	4.8	18.7	21.4	35.3	19.8
18. Increased patients expectations	9,196	9.6	15.6	24.9	32.9	17.0
19. Lack of framework on using e-mail between doctors and patients	9,196	10.6	10.3	15.9	36.5	26.7
20. Lack of remuneration for additional work answering patients e-mails	9,196	7.1	9.0	11.5	30.0	42.4
21. Difficult to use	9,196	5.6	22.3	30.0	30.9	11.2

* 0= I don’t know; 1=Strongly disagree; 2=Somewhat disagree; 3=Somewhat agree; 4=Strongly agree.

Source: Own elaboration.
