# Supplementary material for: Modeling and Predicting Outcomes of eHealth Usage by European Physicians: Multidimensional Approach from a Survey of 9196 General Practitioners
Source: J Med Internet Res. 2018 Oct 22;20(10):e279. doi: 10.2196/jmir.9253 (PMC6231736; doi:10.2196/jmir.9253)
Supplement: Multimedia Appendix 5 [file jmir_v20i10e279_app5.pdf]

Appendix 5a. Personal Health Records (PHR) usage by European general practitioners descriptive statistics. 2012-2013

	N	Mean	Std. Dev.	Minimum	Maximum	Skewness	Kurtosis
22. Request referrals	9,196	0.06	0.234	0	1	3.784	12.322
23. Request appointments	9,196	0.14	0.345	0	1	2.054	2.219
24. Request renewals or prescriptions	9,196	0.10	0.305	0	1	2.598	4.748
25. View their medical records	9,196	0.03	0.163	0	1	5.778	31.394
26. Supplement their medical records	9,196	0.02	0.140	0	1	6.837	44.754
27. View test results	9,196	0.04	0.198	0	1	4.631	19.447

Source: Own elaboration.

Appendix 5b. Personal Health Records (PHR) usage by European general practitioners frequency statistics. 2012-2013

		Valid percentage*	
	N	0	1
22. Request referrals	9,196	94.2	5.8
23. Request appointments	9,196	85.8	14.2
24. Request renewals or prescriptions	9,196	89.6	10.4
25. View their medical records	9,196	97.2	2.8
26. Supplement their medical records	9,196	98.0	2.0
27. View test results	9,196	95.9	4.1

* 0= Not use or not availability; 1=Use.

Source: Own elaboration.
