# Supplementary material for: Modeling and Predicting Outcomes of eHealth Usage by European Physicians: Multidimensional Approach from a Survey of 9196 General Practitioners
Source: J Med Internet Res. 2018 Oct 22;20(10):e279. doi: 10.2196/jmir.9253 (PMC6231736; doi:10.2196/jmir.9253)
Supplement: Multimedia Appendix 6 [file jmir_v20i10e279_app6.pdf]

Appendix 6a. Telehealth usage by European general practitioners descriptive statistics. 2012-2013

	N	Mean	Std. Dev.	Minimum	Maximum	Skewness	Kurtosis
28. Training and education	9,196	0.35	0.478	0	1	0.626	-1.608
29. Consultations with others healthcare practitioners	9,196	0.16	0.367	0	1	1.859	1.456
30. Consultations with patients	9,196	0.09	0.281	0	1	2.951	6.710
31. Monitoring patients remotely at their homes	9,196	0.02	0.136	0	1	7.063	47.897

Source: Own elaboration.

Appendix 6b. Telehealth usage by European general practitioners frequency statistics. 2012-2013

		Valid percentage*	
	N	0	1
28. Training and education	9,196	64.9	35.1
29. Consultations with others healthcare practitioners	9,196	84.0	16.0
30. Consultations with patients	9,196	91.4	8.6
31. Monitoring patients remotely at their homes	9,196	98.1	1.9

* 0= Not use or not availability; 1=Use.

Source: Own elaboration.
