# Supplementary material for: Modeling and Predicting Outcomes of eHealth Usage by European Physicians: Multidimensional Approach from a Survey of 9196 General Practitioners
Source: J Med Internet Res. 2018 Oct 22;20(10):e279. doi: 10.2196/jmir.9253 (PMC6231736; doi:10.2196/jmir.9253)
Supplement: Multimedia Appendix 7 [file jmir_v20i10e279_app7.pdf]

Appendix 7a. Health Information Exchanges (HIE) usage by European general practitioners descriptive statistics. 2012-2013

	N	Mean	Std. Dev.	Minimum	Maximum	Skewness	Kurtosis
32. Interact with patients by e-mail about health-related issues	9,196	0.34	0.472	0	1	0.697	-1.515
33. Patients appointment request	9,196	0.38	0.486	0	1	0.479	-1.771
34. Make appointments at other care providers on patient’s behalf	9,196	0.25	0.431	0	1	1.179	-0.611
35. Send/receive referral and discharge letters	9,196	0.45	0.498	0	1	0.190	-1.964
36. Order supplies for medical practice	9,196	0.28	0.448	0	1	0.986	-1.028
37. Transfer prescriptions to pharmacists	9,196	0.37	0.481	0	1	0.559	-1.688
38. Exchange medical patient data with other health providers or professionals	9,196	0.36	0.480	0	1	0.586	-1.657
39. Receive laboratory reports	9,196	0.69	0.461	0	1	-0.833	-1.307
40. Receive, send and share laboratory reports with other providers or professionals	9,196	0.36	0.479	0	1	0.594	-1.647
41. Exchange patient medication lists with other healthcare professionals or providers	9,196	0.30	0.457	0	1	0.884	-1.218
42. Exchange radiology reports with other healthcare professionals or providers	9,196	0.28	0.451	0	1	0.963	-1.073
43. Exchange medical patient data with any healthcare provider in other countries	9,196	0.10	0.295	0	1	2.741	5.513
44. Certify sick leaves	9,196	0.51	0.500	0	1	-0.051	-1.998
45. Certify disabilities	9,196	0.33	0.469	0	1	0.744	-1.447
46. Exchange administrative patient data with reimbursers or other care providers	9,196	0.27	0.446	0	1	-1.008	-0.984

Source: Own elaboration.

Appendix 7b. Health Information Exchanges (HIE) usage by European general practitioners frequency statistics. 2012-2013

		Valid percentage*	
	N	0	1
32. Interact with patients by e-mail about health-related issues	9,196	66.4	33.6
33. Patients appointment request	9,196	61.6	38.4
34. Make appointments at other care providers on patient’s behalf	9,196	75.4	24.6
35. Send/receive referral and discharge letters	9,196	54.7	45.3
36. Order supplies for medical practice	9,196	72.1	27.9
37. Transfer prescriptions to pharmacists	9,196	63.5	36.5
38. Exchange medical patient data with other health providers or professionals	9,196	64.0	36.0
39. Receive laboratory reports	9,196	30.8	69.2
40. Receive, send and share laboratory reports with other providers or professionals	9,196	64.2	35.8
41. Exchange patient medication lists with other healthcare professionals or providers	9,196	70.2	29.8
42. Exchange radiology reports with other healthcare professionals or providers	9,196	71.7	28.3
43. Exchange medical patient data with any healthcare provider in other countries	9,196	90.4	9.6
44. Certify sick leaves	9,196	48.7	51.3
45. Certify disabilities	9,196	67.4	32.6
46. Exchange administrative patient data with reimbursers or other care providers	9,196	72.5	27.5

* 0= Not use or not availability; 1=Use.

Source: Own elaboration.
