# Supplementary material for: Modeling and Predicting Outcomes of eHealth Usage by European Physicians: Multidimensional Approach from a Survey of 9196 General Practitioners
Source: J Med Internet Res. 2018 Oct 22;20(10):e279. doi: 10.2196/jmir.9253 (PMC6231736; doi:10.2196/jmir.9253)
Supplement: Multimedia Appendix 8 [file jmir_v20i10e279_app8.pdf]

Appendix 8a. Electronic Health Records_ Decisions Support System (EHR_DSS) usage by European general practitioners descriptive statistics. 2012-2013

	N	Mean	Std. Dev.	Minimum	Maximum	Skewness	Kurtosis
47. Clinical guidelines and best practices (i.e. alerts, prompts)	9,196	0.30	0.460	0	1	0.848	-1.281
48. Drug-drug interactions	9,196	0.51	0.499	0	1	-0.044	-1.998
49. Drug-allergy alerts	9,196	0.55	0.498	0	1	-0.187	-1.966
50. Drug-laboratory interactions	9,196	0.24	0.429	0	1	1.193	-0.577
51. Contraindications (i.e. based on age, gender, pregnancy status)	9,196	0.36	0.479	0	1	0.599	-1.641
52. Alert to a critical laboratory value	9,196	0.42	0.494	0	1	0.325	-1.895

Source: Own elaboration.

Appendix 8b. Electronic Health Records_Decisions Support System (EHR_DSS) usage by European general practitioners frequency statistics. 2012-2013

		Valid percentage*	
	N	0	1
47. Clinical guidelines and best practices (i.e. alerts, prompts)	9,196	69.5	30.5
48. Drug-drug interactions	9,196	48.9	51.1
49. Drug-allergy alerts	9,196	45.4	54.6
50. Drug-laboratory interactions	9,196	75.6	24.4
51. Contraindications (i.e. based on age, gender, pregnancy status)	9,196	64.4	35.6
52. Alert to a critical laboratory value	9,196	58.0	42.0

* 0= Not use or not availability; 1=Use.

Source: Own elaboration.
