# Supplementary material for: Modeling and Predicting Outcomes of eHealth Usage by European Physicians: Multidimensional Approach from a Survey of 9196 General Practitioners
Source: J Med Internet Res. 2018 Oct 22;20(10):e279. doi: 10.2196/jmir.9253 (PMC6231736; doi:10.2196/jmir.9253)
Supplement: Multimedia Appendix 9 [file jmir_v20i10e279_app9.pdf]

Appendix 9a. Electronic Health Records_ Data (EHR_DAT) usage by European general practitioners descriptive statistics. 2012-2013

	N	Mean	Std. Dev.	Minimum	Maximum	Skewness	Kurtosis
53. Basic medical parameters (i.e. allergies)	9,196	0.81	0.390	0	1	-1.601	0.563
54. Vital signs	9,196	0.73	0.443	0	1	-1.041	-0.916
55. Treatment outcomes	9,196	0.75	0.435	0	1	-1.134	-0.714
56. Problem list / diagnoses	9,196	0.81	0.389	0	1	-1.614	0.604
57. Medication list	9,196	0.84	0.367	0	1	-1.853	1.435
58. Immunizations	9,196	0.78	0.412	0	1	-1.378	-0.102
59. Medical history	9,196	0.81	0.390	0	1	-1.605	0.578
60. Patient demographics	9,196	0.68	0.467	0	1	-0.764	-1.417
61. Laboratory test results	9,196	0.81	0.393	0	1	-1.572	0.471
62. Radiology test reports	9,196	0.67	0.471	0	1	-0.708	-1.499
63. Radiology test images	9,196	0.30	0.458	0	1	0.876	-1.232
64. Symptoms (reported by patient)	9,196	0.78	0.415	0	1	-1.342	-0.198
65. Reason for appointment	9,196	0.77	0.422	0	1	-1.274	-0.377
66. Clinical notes	9,196	0.79	0.405	0	1	-1.448	0.096
67. Prescriptions / medications	9,196	0.85	0.360	0	1	-1.929	1.723
68. Ordered tests	9,196	0.75	0.431	0	1	-1.172	-0.627
69. Create / update disease management / care plan (i.e. diabetes)	9,196	0.65	0.477	0	1	-0.627	-1.607
70. Finances / billing	9,196	0.45	0.498	0	1	0.180	-1.968
71. Administrative patient data	9,196	0.76	0.425	0	1	-1.240	-0.464

Source: Own elaboration.

Appendix 9b. Electronic Health Records_Data (EHR_DAT) usage by European general practitioners frequency statistics. 2012-2013

		Valid percentage*	
	N	0	1
53. Basic medical parameters (i.e. allergies)	9,196	18.8	81.2
54. Vital signs	9,196	26.9	73.1
55. Treatment outcomes	9,196	25.3	74.7
56. Problem list / diagnoses	9,196	18.6	81.4
57. Medication list	9,196	16.0	84.0
58. Immunizations	9,196	21.6	78.4
59. Medical history	9,196	18.7	81.3
60. Patient demographics	9,196	32.2	67.8
61. Laboratory test results	9,196	19.1	80.9
62. Radiology test reports	9,196	33.3	66.7
63. Radiology test images	9,196	70.1	29.9
64. Symptoms (reported by patient)	9,196	22.1	77.9
65. Reason for appointment	9,196	23.1	76.9
66. Clinical notes	9,196	20.7	79.3
67. Prescriptions / medications	9,196	15.3	84.7
68. Ordered tests	9,196	24.7	75.3
69. Create / update disease management / care plan (i.e. diabetes)	9,196	35.0	65.0
70. Finances / billing	9,196	54.5	45.5
71. Administrative patient data	9,196	23.7	76.3

* 0= Not use or not availability; 1=Use. Source: Own elaboration.
