# Supplementary material for: Modeling and Predicting Outcomes of eHealth Usage by European Physicians: Multidimensional Approach from a Survey of 9196 General Practitioners
Source: J Med Internet Res. 2018 Oct 22;20(10):e279. doi: 10.2196/jmir.9253 (PMC6231736; doi:10.2196/jmir.9253)
Supplement: Multimedia Appendix 10 [file jmir_v20i10e279_app10.pdf]

Appendix 10a. Outcomes of internal practices in eHealth usage by European general practitioners descriptive statistics. 2012-2013

	N	Mean	Std. Dev.	Minimum	Maximum	Skewness	Kurtosis
72. Useful for my practice	9,196	3.38	0.942	0	4	-1.978	4.024
73. Increases the number of patients I can see on average during working hours	9,196	2.41	1.238	0	4	-0.290	-0.981
74. Enhances effectiveness of job	9,196	3.03	1.094	0	4	-1.187	0.789
75. Increases quality of care	9,196	2.95	1.133	0	4	-1.107	0.497
76. Easy to use	9,196	2.88	1.081	0	4	-0.989	0.474
77. Easy to get it to do what I want	9,196	2.70	1.118	0	4	-0.747	-0.092
78. Flexible to use / interact with	9,196	2.68	1.143	0	4	-0.786	-0.065
79. Colleagues who are important to me think I should use ICT systems	9,196	2.24	1.494	0	4	-0.325	-1.347
80. People who influence my behaviour think I should use ICT systems	9,196	2.18	1.477	0	4	-0.258	-1.363
81. People who influence my clinical behaviour think I should use ICT systems	9,196	2.19	1.474	0	4	-0.268	-1.354
82. I have necessary resources to use ICT systems	9,196	2.83	1.163	0	4	-0.938	0.054
83. I have knowledge to use ICT systems	9,196	2.94	1.062	0	4	-1.119	0.835
84. I have technical assistance available	9,196	2.84	1.138	0	4	-0.862	-0.069
85. Using ICT systems is entirely under my control	9,196	2.49	1.163	0	4	-0.410	-0.736

Source: Own elaboration.

Appendix 10b. Outcomes of internal practices in eHealth usage by European general practitioners frequency statistics. 2012-2013

		Valid percentage*				
	N	0	1	2	3	4
72. Useful for my practice	9,196	3.5	2.1	5.3	31.1	58.0
73. Increases the number of patients I can see on average during working hours	9,196	7.3	19.3	22.6	27.3	23.5
74. Enhances effectiveness of job	9,196	4.6	6.5	11.6	36.4	40.9
75. Increases quality of care	9,196	5.5	7.3	12.1	36.9	38.2
76. Easy to use	9,196	4.8	6.5	16.7	39.7	32.4
77. Easy to get it to do what I want	9,196	5.6	9.1	20.9	38.3	26.1
78. Flexible to use / interact with	9,196	6.8	8.4	20.3	38.8	25.7
79. Colleagues who are important to me think I should use ICT systems	9,196	21.2	12.8	13.1	26.8	26.2
80. People who influence my behaviour think I should use ICT systems	9,196	21.0	14.6	13.6	26.8	24.0
81. People who influence my clinical behaviour think I should use ICT systems	9,196	20.9	14.4	13.7	27.0	24.0
82. I have necessary resources to use ICT systems	9,196	6.0	9.2	13.8	37.6	33.4
83. I have knowledge to use ICT systems	9,196	4.7	5.9	13.6	42.1	33.8
84. I have technical assistance available	9,196	4.7	10.2	15.4	36.2	33.5
85. Using ICT systems is entirely under my control	9,196	5.6	16.5	23.0	33.1	21.8

* 0= I don’t know; 1=Strongly disagree; 2=Somewhat disagree; 3=Somewhat agree; 4=Strongly agree.

Source: Own elaboration.
