# Supplementary material for: Modeling and Predicting Outcomes of eHealth Usage by European Physicians: Multidimensional Approach from a Survey of 9196 General Practitioners
Source: J Med Internet Res. 2018 Oct 22;20(10):e279. doi: 10.2196/jmir.9253 (PMC6231736; doi:10.2196/jmir.9253)
Supplement: Multimedia Appendix 11 [file jmir_v20i10e279_app11.pdf]

Appendix 11a. Outcomes of practices with patients in eHealth usage by European general practitioners descriptive statistics. 2012-2013

	N	Mean	Std. Dev.	Minimum	Maximum	Skewness	Kurtosis
86. Reduce medical errors	9,196	2.76	1.116	0	4	-0.924	0.126
87. Improvement in the quality of diagnosis decisions	9,196	2.77	1.132	0	4	-0.913	0.165
88. Improvement in the quality of treatment	9,196	2.79	1.134	0	4	-0.948	0.209
89. Enhance self-evaluation	9,196	2.71	1.222	0	4	-0.894	-0.112
90. More data for clinical research and public health	9,196	3.07	1.095	0	4	-1.418	1.520
91. Facilitate patients' education and adherence to prescriptions	9,196	2.70	1.171	0	4	-0.832	-0.083
92. Improvement in patients' satisfaction	9,196	2.55	1.237	0	4	-0.737	-0.384
93. Increased patients' access to healthcare (i.e. booking online appointment, viewing data)	9,196	2.67	1.190	0	4	-0.783	-0.228
94. Avoid unnecessary test and duplications	9,196	3.01	1.079	0	4	-1.172	0.829
95. Increase average number of patients receiving help during one day	9,196	2.45	1.207	0	4	-0.395	-0.776
96. Reduce pharmaceutical expenditure	9,196	2.38	1.277	0	4	-0.452	-0.839
97. Shorter waiting lists	9,196	2.31	1.235	0	4	-0.294	-0.869
98. Allow more efficient consultations	9,196	2.78	1.147	0	4	-0.915	0.087
99. Improvement in coordination between the different levels of the health system	9,196	2.94	1.110	0	4	-1.195	0.865
100. Expedite workflow due to the availability of patients' clinical data	9,196	2.96	1.110	0	4	-1.144	0.689
101. Improvement in the efficiency of the whole health system	9,196	2.97	1.109	0	4	-1.179	0.801

Source: Own elaboration.

Appendix 11b. Outcomes of practices with patients in eHealth usage by European general practitioners frequency statistics. 2012-2013

		Valid percentage*				
	N	0	1	2	3	4
86. Reduce medical errors	9,196	7.1	7.8	15.9	40.8	28.4
87. Improvement in the quality of diagnosis decisions	9,196	6.3	8.1	16.4	41.2	28.1
88. Improvement in the quality of treatment	9,196	6.2	8.2	14.9	41.5	29.2
89. Enhance self-evaluation	9,196	9.2	8.0	14.7	39.3	28.8
90. More data for clinical research and public health	9,196	6.0	4.0	8.7	39.9	41.4
91. Facilitate patients' education and adherence to prescriptions	9,196	7.4	8.6	17.7	39.2	27.0
92. Improvement in patients' satisfaction	9,196	10.8	8.4	19.0	38.6	23.2
93. Increased patients' access to healthcare (i.e. booking online appointment, viewing data)	9,196	7.7	9.5	17.8	37.9	27.1
94. Avoid unnecessary tests and duplications	9,196	4.5	6.4	11.9	38.6	38.6
95. Increase average number of patients receiving help during one day	9,196	7.5	15.3	24.6	29.9	22.7
96. Reduce pharmaceutical expenditure	9,196	11.6	13.4	21.9	31.5	21.6
97. Shorter waiting lists	9,196	9.8	16.3	26.4	27.7	19.8
98. Allow more efficient consultations	9,196	6.3	9.0	14.7	40.6	29.4
99. Improvement in coordination between the different levels of the health system	9,196	6.1	5.7	11.4	41.9	35.0
100. Expedite workflow due to the availability of patients' clinical data	9,196	5.3	6.4	12.4	38.4	37.5
101. Improvement in the efficiency of the whole health system	9,196	5.6	5.9	12.0	39.3	37.2

* 0= I don't know; 1=Strongly disagree; 2=Somewhat disagree; 3=Somewhat agree; 4=Strongly agree.

Source: Own elaboration.
