# Supplementary material for: Modeling and Predicting Outcomes of eHealth Usage by European Physicians: Multidimensional Approach from a Survey of 9196 General Practitioners
Source: J Med Internet Res. 2018 Oct 22;20(10):e279. doi: 10.2196/jmir.9253 (PMC6231736; doi:10.2196/jmir.9253)
Supplement: Multimedia Appendix 12 [file jmir_v20i10e279_app12.pdf]

Multimedia Appendix 12. Explanatory factors of some dimensions of European general practitioners' eHealth usage and eHealth usage outcomes in 2012-2013.

Dimension/variable		Standardized coefficient	*P* value	Error	*P* value
**1. ICT usage (ICTUS)**				0.028	<.001
1.	Internet usage during consultations	0.245	<.001	0.443	<.001
2.	Computer usage in general practice	0.064	<.001	0.003	<.001
3.	Computer usage to display patients' health-related information	0.287	<.001	0.441	<.001
4.	Problems of compatibility in electronically exchanging patient data	0.481	<.001	1.215	<.001
5.	Medical organizations in contact with general practitioner	0.640	<.001	2.840	<.001
Goodness-of-fit indices: NFI[b]: 0.996; RFI[c]: 0.984; IFI[d]: 0.997; TLI[e]: 0.990; CFI[f]: 0.997; RMSEA[g]: 0.012					
**2. Barriers to eHealth usage (BARRIERS)**				0.513	<.001
6.	Lack of financial incentives	0.607	<.001	0.881	<.001
7.	Lack of financial resources	0.632	<.001	0.821	<.001
8.	Lack of access to technology	0.631	<.001	0.797	<.001
9.	Lack of technical support	0.649	<.001	0.748	<.001
10.	Lack of interoperability and standards	0.597	<.001	1.084	<.001
11.	Lack of sufficient resilience –ICT[h] systems can fail	0.653	<.001	0.788	<.001
12.	Lack of sufficient security and risk control	0.643	<.001	0.833	<.001
13.	Lack of framework on confidentiality and privacy issues	0.655	<.001	0.874	<.001
14.	Lack of time – additional workload	0.681	<.001	0.687	<.001
15.	Lack of sufficient ICT skills among general practitioners	0.663	<.001	0.628	<.001
16.	Lack of sufficient training for healthcare professionals	0.673	<.001	0.605	<.001
17.	Lack of clear motivation for using ICT	0.623	<.001	0.798	<.001
18.	Increased patient expectations	0.528	<.001	1.042	<.001
19.	Lack of framework on using e-mail for communication between doctors and patients	0.574	<.001	1.089	<.001
20.	Lack of remuneration for additional work answering patients e-mails	0.548	<.001	1.069	<.001
21.	Difficult to use	0.629	<.001	0.703	<.001
Goodness-of-fit indices: NFI: 0.984; RFI: 0.975; IFI: 0.985; TLI: 0.977; CFI: 0.985; RMSEA: 0.040					
**8. eHealth usage outcomes in internal practices (OUTINTPRA)**				0.456	<.001
72.	Useful for my practice	0.717	<.001	0.430	<.001
73.	Increases the number of patients I can see on average during working hours	0.624	<.001	0.936	<.001
74.	Enhances effectiveness of job	0.735	<.001	0.548	<.001
75.	Increases quality of care	0.686	<.001	0.679	<.001
76.	Easy to use	0.801	<.001	0.419	<.001
77.	Easy to get it to do what I want	0.769	<.001	0.511	<.001
78.	Flexible to use / interact with	0.744	<.001	0.583	<.001
79.	Colleagues who are important to me think I should use ICT systems	0.477	<.001	1.725	<.001
80.	People who influence my behavior think I should use ICT systems	0.442	<.001	1.754	<.001
81.	People who influence my clinical behavior think I should use ICT systems	0.435	<.001	1.762	<.001
82.	I have the necessary resources to use ICT systems	0.628	<.001	0.819	<.001
83.	I have the knowledge to use ICT systems	0.627	<.001	0.681	<.001
84.	I have technical assistance available	0.558	<.001	0.887	<.001
85.	Using ICT systems is entirely under my control	0.616	<.001	0.838	<.001
Goodness-of-fit indices: NFI: 0.991; RFI: 0.986; IFI: 0.992; TLI: 0.987; CFI: 0.992; RMSEA: 0.037					
**9. eHealth usage outcomes in practices with patients (OUTPRAPAT)**				0.730	<.001

	86.	Reduce medical errors	0.740	<.001	0.604	<.001
	87.	Improvement in the quality of diagnosis decisions	0.783	<.001	0.495	<.001
	88.	Improvement in the quality of treatment	0.784	<.001	0.495	<.001
	89.	Enhance self-evaluation	0.696	<.001	0.769	<.001
	90.	More data for clinical research and public health	0.665	<.001	0.669	<.001
	91.	Facilitate patients' education and adherence to prescriptions	0.723	<.001	0.652	<.001
	92.	Improvement in patient satisfaction	0.707	<.001	0.759	<.001
	93.	Increased patient access to healthcare (e.g., booking online appointments, viewing data)	0.647	<.001	0.823	<.001
	94.	Avoid unnecessary tests and duplications	0.710	<.001	0.577	<.001
	95.	Increase average number of patients receiving help during one day	0.697	<.001	0.749	<.001
	96.	Reduce pharmaceutical expenditure	0.649	<.001	0.945	<.001
	97.	Shorter waiting lists	0.677	<.001	0.823	<.001
	98.	Allow more efficient consultations	0.767	<.001	0.541	<.001
	99.	Improvement in coordination between the different levels of the healthcare system	0.720	<.001	0.592	<.001
	100.	Expedite workflow due to the availability of patients' clinical data	0.741	<.001	0.555	<.001
	101.	Improvement in the efficiency of the whole healthcare system	0.797	<.001	0.448	<.001
	Goodness-of-fit indices: NFI: 0.989; RFI: 0.983; IFI: 0.990; TLI: 0.984; CFI: 0.990; RMSEA: 0.038					

[a]Regression analysis: structural equation modeling (SEM); direct effects.

[b]NFI: normed fit index

[c]RFI: relative fit index.

[d]IFI: incremental fit index.

[e]TLI: Tucker-Lewis index

[f]CFI: comparative fit index.

[g]RMSEA: root mean square error of approximation

[h]ICT: information and communication technology
