# Supplementary material for: Modeling and Predicting Outcomes of eHealth Usage by European Physicians: Multidimensional Approach from a Survey of 9196 General Practitioners
Source: J Med Internet Res. 2018 Oct 22;20(10):e279. doi: 10.2196/jmir.9253 (PMC6231736; doi:10.2196/jmir.9253)
Supplement: Multimedia Appendix 13 [file jmir_v20i10e279_app13.pdf]

Appendix 12. eHealth usage by European general practitioners dimensions frequency statistics*. 2012-2013

		Valid percentage																			
	N	0	1	2	3	4	5	6	7	8	9	10	11	12	13	14	15	16	17	18	19
3. Personal Health Records (PHR)	9,196	62.0	14.9	11.8	6.4	2.1	1.2	1.6	-	-	-	-	-	-	-	-	-	-	-	-	-
4. TeleHealth	9,196	58.1	27.3	10.3	3.4	0.8	-	-	-	-	-	-	-	-	-	-	-	-	-	-	-
5. Health Information Exchanges (HIE)	9,196	12.0	7.6	10.1	9.9	9.8	8.3	7.6	6.6	6.0	5.1	4.8	4.1	2.8	2.1	1.8	1.7	-	-	-	-
6. Electronic Health Records_Decisions Support Systems (HER_DSS)	9,196	29.8	13.0	12.5	12.4	10.8	9.3	12.3	-	-	-	-	-	-	-	-	-	-	-	-	-
7. Electronic Health Records_Data (HER_DAT)	9,196	10.9	0.7	0.7	0.7	1.0	0.8	0.9	0.8	1.1	1.2	1.3	1.8	2.3	3.1	4.3	7.5	11.8	19.0	19.4	10.7

* Number of practices in each dimension.

Source: Own elaboration.
